# Hyaenas and early humans in the latest Early Pleistocene of South-Western Europe

**DOI:** 10.1038/s41598-021-03547-7

**Published:** 2021-12-15

**Authors:** Gonzalo J. Linares-Matás, Norman Fernández Ruiz, María Haber Uriarte, Mariano López Martínez, Michael J. Walker

**Affiliations:** 1 St. Hugh’s College, University of Oxford, Oxford, OX2 6LE UK; 2 Murcian Association for the Study of Palaeoanthropology and the Quaternary (MUPANTQUAT), Murcia, Spain; 3 Department of Prehistory, Archaeology, Ancient History, Mediaeval History and Historiographical Studies, Faculty of Letters, University of Murcia, La Merced Campus, 30001 Murcia, Spain; 4 Department of Zoology and Physical Anthropology, Faculty of Biology, University of Murcia, Espinardo Campus, 30100 Murcia, Spain

**Keywords:** Archaeology, Palaeontology

## Abstract

Throughout the Pleistocene, early humans and carnivores frequented caves and large rock-shelters, usually generating bone accumulations. The well-preserved late Early Pleistocene sedimentary sequence at Cueva Negra del Estrecho del Río Quípar (CNERQ) has provided substantial evidence concerning the behavioural and adaptive skills of early humans in Western Europe, such as butchery practices, lithic technology or tending fire, whilst also bearing witness to the bone-altering activities of carnivores. Recent fieldwork has allowed the re-examination of the spatial and taphonomical nature of the macrofaunal assemblage from the upper layers of Complex 2. These layers are somewhat different from most of the underlying sequence, in showing quite a high representation of cranial and post-cranial bones of large mammals, including several *Megaloceros* *carthaginiensis* antlers. The presence of *Crocuta* sp. at Cueva Negra represents one of the earliest instances of this genus in Western Eurasia. Identification of several juvenile *Crocuta* sp. remains alongside coprolites and bones with carnivore damage, indicates sporadical hyaenid denning activity. Furthermore, the presence of bones with percussion and cut-marks near to several hammerstones suggests a clear albeit limited anthropogenic input. We interpret the available taphonomical and spatial evidence from these layers as reflecting a multi-patterned palimpsest, likely representing the non-simultaneous and short-lived co-existence of hyaenas, humans, and other small carnivores in the Cueva Negra palaeolandscape during the final phase of sedimentation preserved at the site.

## Introduction

Co-ocurrence of early humans and large carnivores within the same ecosystem is well documented for Pleistocene landscapes^1–4^. From a biogeographical standpoint, the late Early Pleistocene was a dynamic period in Europe, encompassing the arrival of early human groups and noteworthy changes in the composition of faunal guilds^5,6^. Early humans were already present in western Mediterranean Europe from around 1.4–1.2 Ma at Pirro Nord^7^, Sima del Elefante TD9 in Atapuerca^8^, Barranco León and Fuente Nueva-3 in Orce^9^, Pont de Lavaud^10^ and Le Vallonet^11^ in France. The biogeographical dispersal of *Crocuta* *sp.* into western Europe during the latest Early Pleistocene, around 0.8 Ma^12^, coincides with series of hominin sites that show evidence of a more sustained occupation of the Western Mediterranean^13–15^. Hyaenids and other large carnivores likely played an important role in the configuration of human dispersal routes from Africa and Asia to Europe during the Early and Middle Pleistocene, according to plausible inferences drawn from considerations of their palaeoecology and dietary adaptations^6,16–18^. Thus, interactions between carnivores and early humans in south-western Europe are an important focus of attention in palaeontological and taphonomical research at both open-air and cave sites, e.g. Barranc de la Boella^4^, Barranco León-D^2^, Bois-de-Riquet^19^, Fuente Nueva-3^2,20^, Atapuerca-Gran Dolina^3,21,22^, and Vallparadís^23^. In order to establish the origin of a dual-patterned archaeological faunal assemblage, it is necessary to integrate taphonomical and spatial data for assessment of the roles of different agents and the nature of site formation processes involved, alongside the potential degree of direct or indirect competition between different consumers by taking into account the temporal sequence of carcass access and the likely biomass available to them^24–26^. In this paper, we assess site formation processes and human-carnivore dynamics at the late Early Pleistocene site of Cueva Negra.

**Figure 1:**
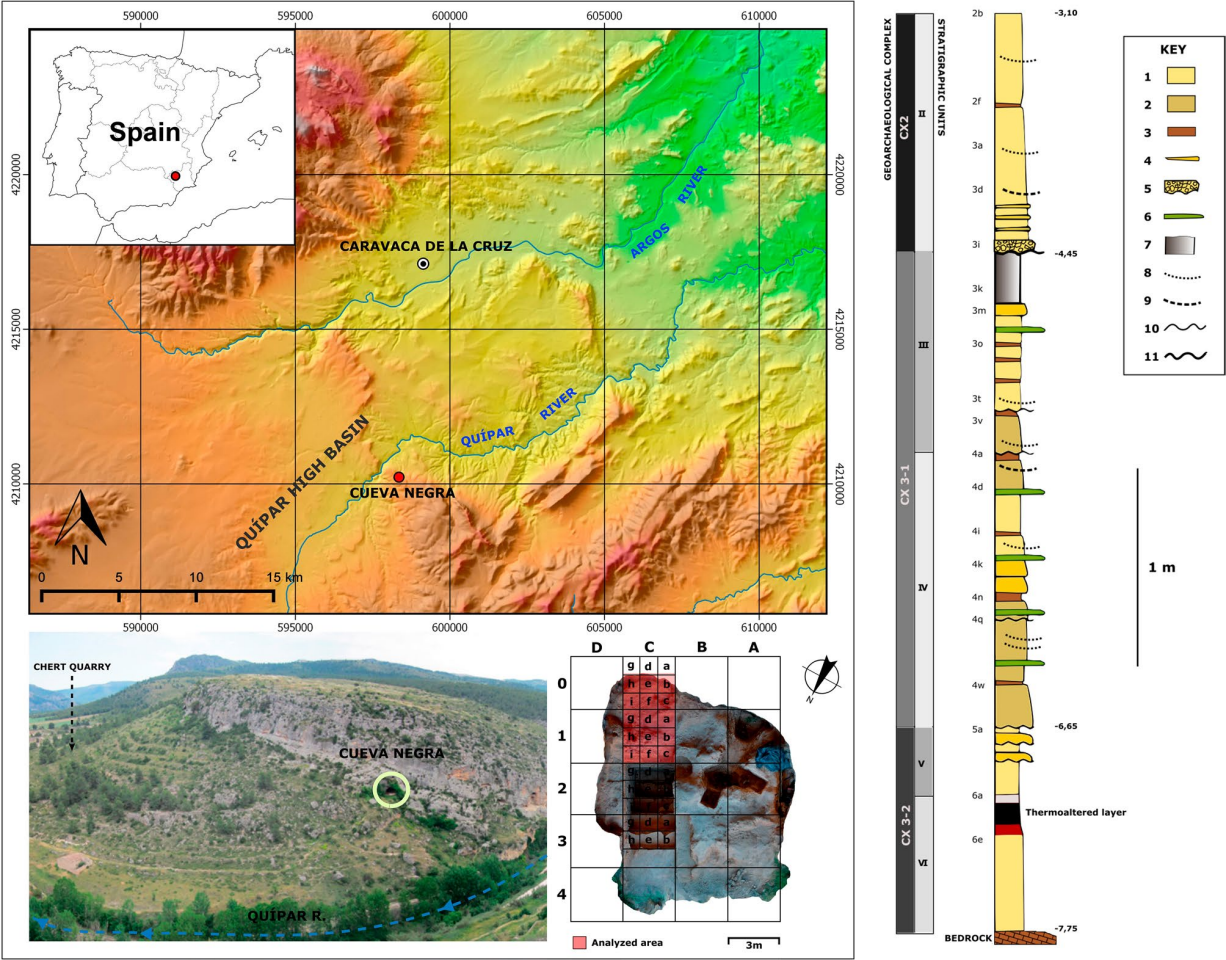
Site location map and excavation grid on orthophoto. Model of the stratigraphic sequence of CNERQ, after Angelucci et al.^35^ (reproduced with permission). Key: 1—silty sand, massive or poorly laminated; 2—silty sand with flat lamination or cross-bedding; 3—silt or clayey silt, massive or with flat lamination; 4—sand; 5—gravel; 6—stone-lines formed of fine granules; 7—fine lenses of granules to fine gravel; 8—calcium carbonate crusts; 9—main erosive surface between Complex 2 and Complex 3; 10—minor erosive surfaces. Panoramic photograph by M.L.M. Map generated using Arcmap, and composition made by N.F.R. using Inkscape.

Cueva Negra del Estrecho del Río Quípar (CNERQ) is a large, north-facing rock-shelter, lying at 740 m a.s.l in a biocalcarenite cliff at the exit of the Río Quípar gorge (“Estrecho”), below the hamlet of La Encarnación, 10 km south of Caravaca de la Cruz, Murcia, SE Spain (38° 02′ 5.8″ N; 1° 53′ 5.8″ W; Fig. 1). Systematic scientific excavations began in 1990. Recent accounts^26–28^ address inaccuracies and shortcomings of some earlier publications. Palaeomagnetic reverse polarity throughout the 5-m depth of sedimentary deposits^29^, micromammalian biostratigraphy^30^, and ESR-dating^31^ indicate a late Early Pleistocene age somewhere between the end of the Jaramillo sub-chron (ca. 0.99 Ma) and the Matuyama-Brunhes boundary (ca. 0.772 Ma). It therefore bears comparison with the age of Atapuerca TD-6^15^. Palynological evidence^32^, the avian taxa identified by Anne Eastham, corroborated by Dr. Anna Rufà Bonache (*pers.* *comm.*), and new, as yet unpublished, herpetofaunal data from Dr. Hugues Blain (*pers.* *comm.*), suggest temperate environmental conditions, compatible with interglacial climates likely during MIS-21 or early MIS-19 (i.e., before 0.772 Ma), since biostratigraphical comparisons of rodent palaeontology seem to favour a time after MIS-23^30^). Thermal alteration characterises bones and chert artefacts and cores excavated in a layer of dark, blackish soil present 4.5 m down, almost at the base of the sedimentary sequence, in Complex 3-2^33,34^ (Supplementary Fig. S1). This layer has been identified over 5–6 square metres to date, though because of its depth and the nature of the excavation process, it has not yet been possible to ascertain its full extent; no hearth-stones, fire-pits, or circumscribed "hearths" have been identified in this context.

**Figure 2:**
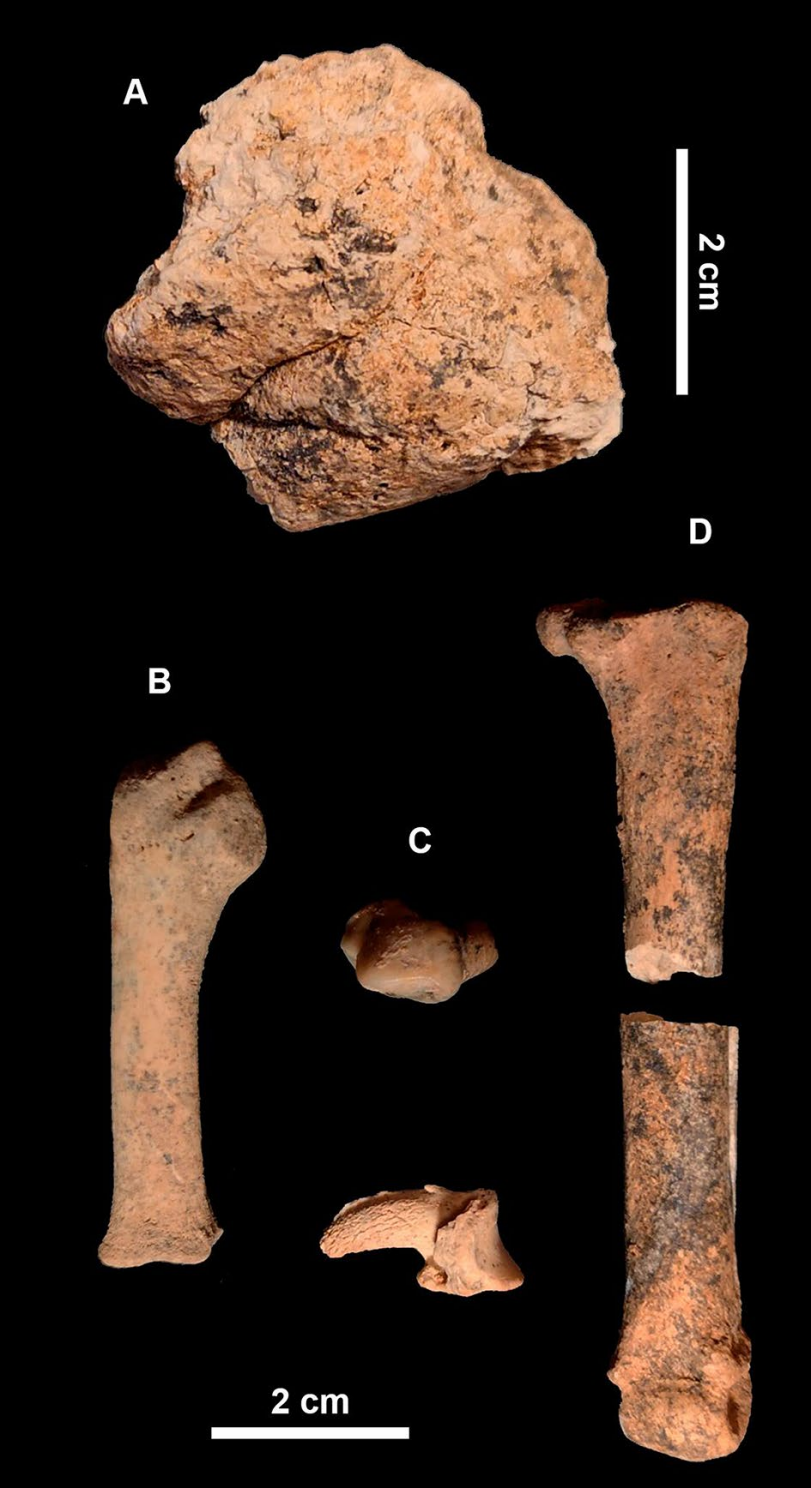
(**A**) Carnivore coprolite retrieved from Cueva Negra upper complex 2 in 2019. (**B**–**D**) Hyaena remains excavated in 2019, from levels 2d–2f of Complex 2 at Cueva Negra. Left: left fifth metacarpal with unfused distal epiphysis; centre: P1 tooth germ; bottom: third phalanx; right: fourth metatarsal broken during excavation. Photographs by Maria Diget Sletterød and initial processing by Alfredo Sánchez, with subsequent post-processing and final composition by N.F.R. Reproduced with permission.

The macrofauna is under examination by Dr. Jan van der Made^27,28^ and includes several taxa that existed at that time, viz., *Megaloceros* *novocarthaginiensis*, *Dama* cf. *vallonnetensis*, *Stephanorhinus* cf. *etruscus*, *Equus* cf. *altidens,* *Bison* sp.; bear teeth, probably of *Ursus* cf. *deningeri*, and fragments of a proboscidean mandible and vertebra, have also been found. A mandibular fragment of *Crocuta* sp. at CNERQ mirrors the presence of the genus at Atapuerca Trinchera Dolina TD-6.3^21^, and the 2019 excavation uncovered more hyaenid fragments at the site (Fig. 2) alongside more fragments of *Megaloceros* *novocarthaginiensis* and *Stephanorhinus* cf. *etruscus*. Other mammals from CNERQ include *Macaca* sp., *Sus* *scrofa*, *Capreolus* sp., a caprine that could be *Hemitragus* *bonali* or *Capra* *alba,* a mustelid, and probably *Lynx* sp.

The fundamentally uniform, homogeneous nature of the CNERQ sedimentary sequence is the outcome of low-energy fluviatile transport that led to no more that scanty horizontal displacement of finds, and is attributed to sporadical, perhaps seasonal, overflow of a swampy lake, beside the rock-shelter, fed by the River Quípar which flows northwards along the active sinistral Quípar Fault^27,35^. Subsequent neotectonic uplift raised the cave above the water table, thereby preventing riverine erosion of the sedimentary accumulation within.

The last phase of that accumulation is represented by sedimentary Complex 2—the focus of this paper—which comprises a truncated alluvial sequence with low porosity, good sedimentary organisation, and alternations of thin beds and lenses, mostly of fine and medium sand-size silty particles mainly of calcareous litharenite (rather than silica sand), with occasional deposits of coarser, less-silty, sand-size particles, and rolled small gravel rarely larger than ~ 5 mm in size; all of which reflect mainly low- and occasionally medium-energy dynamics^35^. Fluviatile rolled gravel between 5 and 50 mm in size is conspicuous by its absence, though within this range there are angular biocalcarenite clasts eroded out of the roof or walls of the rock-shelter; rolled cobbles of greater size, excavated in the sediment and often flaked, owe to Palaeolithic exploitation of an Upper Miocene marine conglomerate outcrop, 0.8 km east of the cave, containing cobbles of chert, quartzite, and hard siliceous limestone. At the microscopical level, three main sedimentary inputs were recognised in terms of lithology, size, and roundness, namely, (1) dominant (sub-)rounded sand-size grains; (2) scarce-to-common angular, coarse biocalcarenite fragments; (3) fragments (mainly chert) of artefacts or manuports, bone, and coprolites^35^.

Excavation in 2019 at the rear of the rock-shelter afforded a new opportunity to study the origin, nature, and spatial organisation of the upper Complex 2 assemblage (Supplementary Fig. S2). Our aim here is to characterise the bone accumulation in the upper layers of the late Early Pleistocene Complex 2 of CNERQ, and, by assessing the nature and distribution of bones with carnivore damage and those with anthropogenic modifications, to investigate whether the primary composition of the accumulation derives primarily from a carnivore-ravaged anthropogenic site, the den of a bone-gathering carnivore, or represents a combination of alternating occupational sequences, with contributions from different agents, which coalesced into a palimpsest.

## Results

### Bone taphonomy

**Figure 3:**
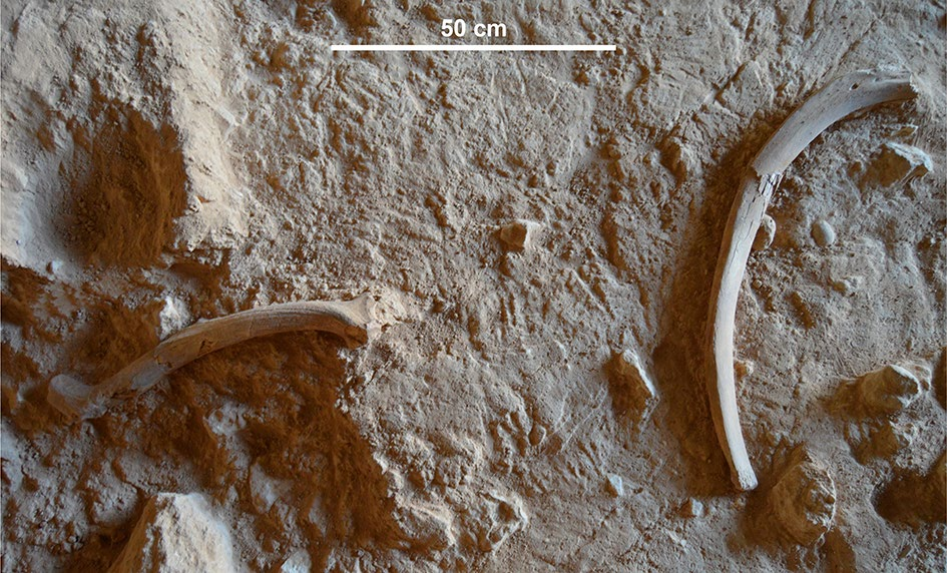
Zenithal view of the excavation of Unit II. Left: gnawed *Megaloceros* antler. Right: rib of very large herbivore, showing gnawed ends and a cluster of several large pits in the proximal end. Note the presence of an in-situ dry fracture. Picture by Pedro Lucas Salcedo, reproduced with permission.

1596 macrofaunal remains were retrieved from levels 2c–2f of CNERQ Complex 2 during the 2019 field season (Fig. 3) and examined from zooarchaeological and taphonomical perspectives.

In terms of skeletal profiles, the upper levels of CNERQ Complex 2 are characterised by a relatively high proportion of cranial fragments, axial bones, and autopodial elements, particularly in relation to the rest of the stratigraphical sequence at the site. Large- and medium-size animals predominate, with a broadly similar representation of small- and very large-size animals (Table 1). The remains belonging to very large-size animals are mostly those of neonate-juvenile individuals, and indeed a high representation of juveniles (including neonates) and subadults was found across almost all species in terms of MNI (Supplementary Table S1). In terms of NR, juvenile and neonate remains across all size classes represent 5.6% (n = 90). An extremely worn lateral incisor of *Ursus* cf. *deningeri* (I3) is the only specimen that clearly identifies an old individual, although the relative paucity of complete dental elements hampers recognition of old specimens.

**Table 1:** Skeletal part distribution with regards to carcass size in the faunal assemblage from the upper levels of Complex 2 at Cueva Negra.

Element NISP	Very large size	Large size	Medium size	Small size	Size Indet	Total NISP/element
*Antler/Horn*	0	7	1	2	7	**17**
*Cranial* *Fragment*	4	11	9	3	74	**101**
*Mandible*	0	0	0	1	0	**1**
*Maxilla*	0	1	1	0	0	**2**
*Isolated* *teeth*	0	4	4	4	17	**29**
*Vertebra*	0	1	5	1	4	**11**
*Rib*	7	16	22	20	7	**72**
*Sterna*	0	0	0	0	0	**0**
*Scapula*	0	1	3	3	2	**9**
*Humerus*	0	3	9	3	1	**16**
*Radius*	0	2	2	0	0	**4**
*Ulna*	0	1	0	0	0	**1**
*Carpal/tarsal*	0	1	3	0	6	**10**
*Metapodial*	0	3	3	7	0	**13**
*Os* *coxae*	1	0	4	1	2	**8**
*Femur*	0	3	4	1	0	**8**
*Patella*	0	0	0	0	0	**0**
*Tibia*	0	3	1	1	0	**5**
*Phalanx*	0	1	7	3	4	**15**
*Sessamoid*	0	2	2	0	1	**5**
*Ephiphysis* *Indet*	8	13	10	1	111	**143**
*Diaphysis* *Indet*	17	73	72	13	155	**330**
*Axial* *Indet*	5	33	26	16	132	**212**
*Indet*	–	–	–	–	584	**584**
*Total* *NISP* *per* *size* *class*	42	179	188	80	1107	1596

The high degree of fragmentation of the assemblage (86.9% of bone fragments are <  ~ 30 mm, and only 0.94% of fragments are >  ~ 100 mm) precludes accurate identification of one-third of the assemblage, and epiphyses, while frequent, were generally represented by lumps of cancellous bone, which limited their taxonomical potential. There is a high incidence of post-depositional dry fractures in relation to green fractures on bone diaphyses (3.3:1, 19.6% of the assemblage). Some rodent gnawing damage was detected on dry and weathered bone fragments (n = 36), with ~ 70% of them being found in the square closest to the cave wall (C0e; n = 25).

**Figure 4:**
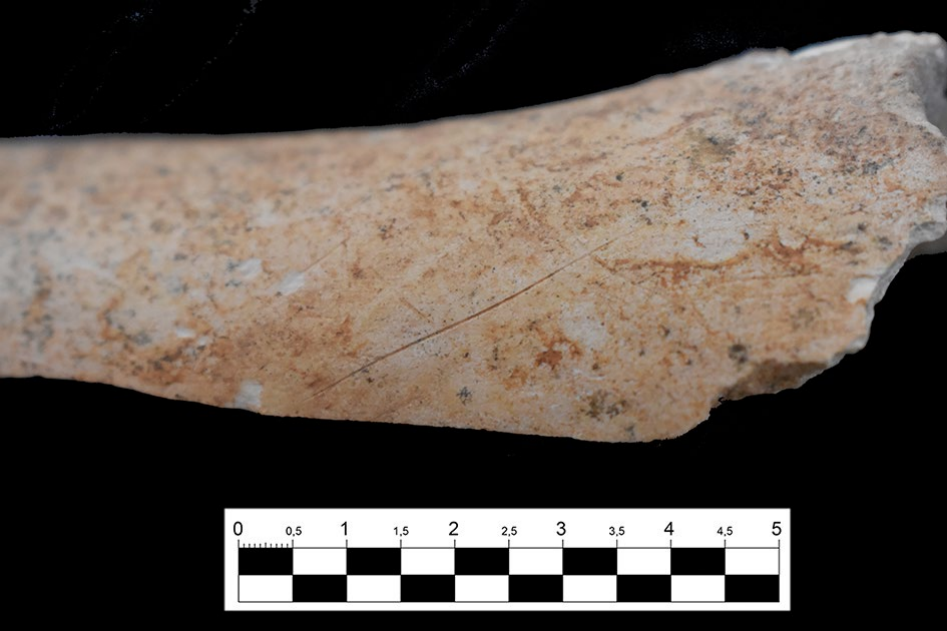
Oblique cut-mark on the diaphysis of an upper limb bone (humerus) from a large-sized herbivore. The bone also presents Mn staining, some polish, and biochemical alterations. Picture by Pedro Lucas Salcedo, reproduced with permission.

Anthropogenic modifications in the form of cut-marks are relatively infrequent in the assemblage (2.5% NR). Furthermore, all cut-marks identified in the upper Complex 2 assemblage were relatively simple incisions, likely generated using sharp unmodified flakes, although geometric morphometric assessments are needed to test this hypothesis and identify the raw material employed in butchery activities^36–38^. In relation to their skeletal part and carcass size distribution, 65% of all cut-marks from this assemblage (n = 26/40) were found on axial or long bone diaphyses of large and medium-sized animals, mostly associated with evisceration and filleting, rather than disarticulation (Fig. 4; Tables S2, S3). Several diaphyseal fragments show impact negatives (Supplementary Fig. S3) and some percussion marks, suggesting intensive exploitation of animal resources, although they are not very frequent. These impact negatives are broad, homogeneous, sometimes overlapping, and likely indicate the use of unmodified hammerstones. No skeletal element ascribed to a juvenile individual showed evidence of anthropogenic modifications.

**Figure 5:**
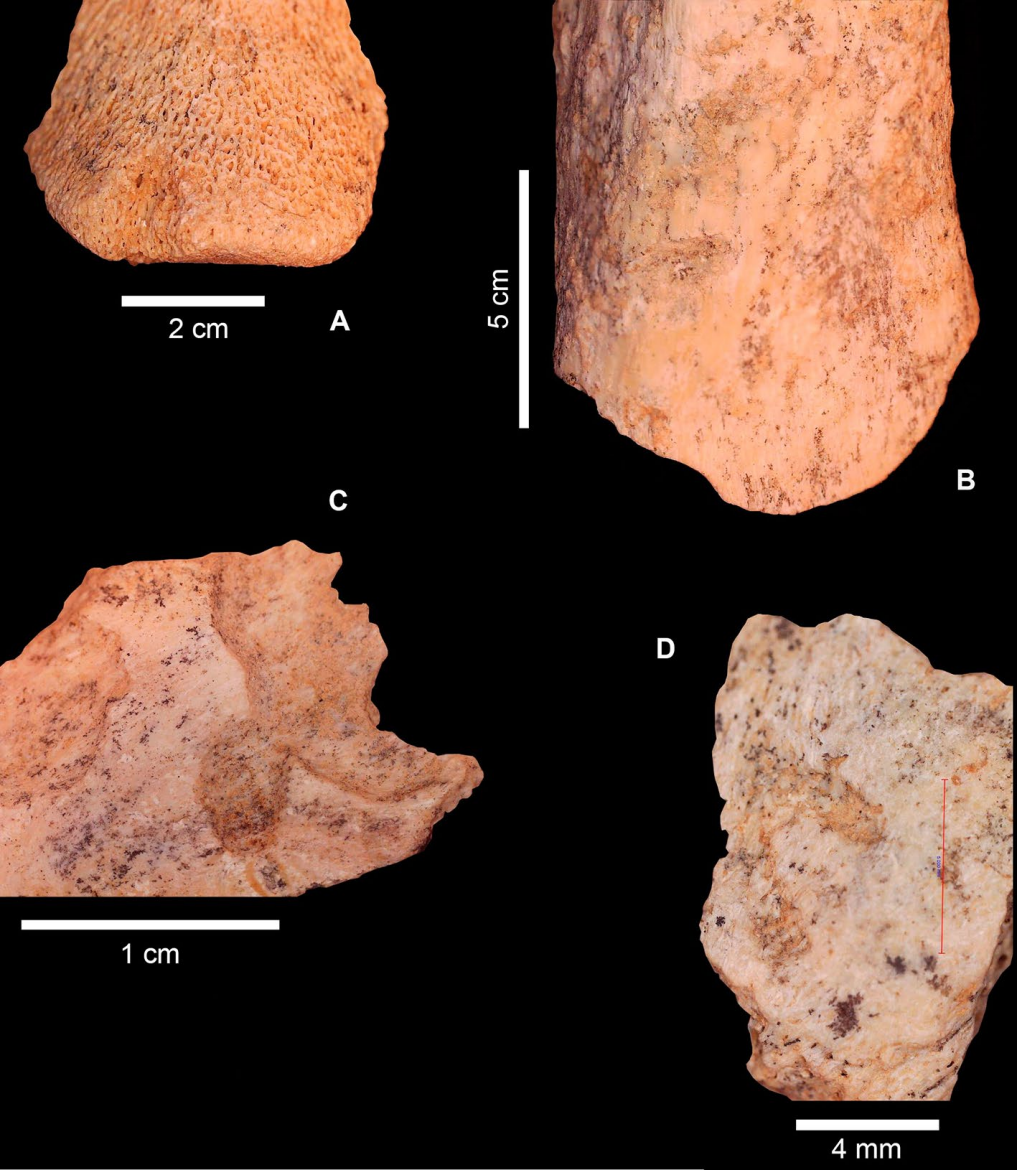
Faunal sample showing several carnivore alterations from the CNERQ upper Complex 2 assemblage. (**A**) Digested cancellous tissue. (**B**) Scores on a tibia shaft fragment of a medium-sized herbivore. (**C**) Digested bone splinter with localised chemical erosions. (**D**) two large scores on a shaft fragment. Pictures by G.L.M. using Celestron Microscan. Processing and composition by N.F.R. using Photoshop and Inkscape.

Tooth-marks (Fig. 5) show a relatively even distribution with regard to carcass size, being most abundant on large- and medium-size prey, although they appear to be over-represented on both small and very large animals (Supplementary Table S2). Among tooth-marks, pits (n = 181, or 11.65% NR) are more frequent than scores (n = 93, 5.8% NR), while punctures are very rare (n = 2). Nonetheless, these percentages are biased owing to intense post-depositional fragmentation and the high proportion of very small fragments; when we consider anatomical part NISP (n = 982), pits increase to 17.92% (n = 176/982), and scores increase to 9.18% (n = 90/982) for this sub-assemblage, which may be more representative of original assemblage composition overall.

Interestingly, 29% of tooth-mark instances (n = 80) were found on elements assigned to juvenile individuals, even though diagnostically juvenile elements represent only 5.6% of the assemblage (7.1% if we remove from consideration the fully skeletally-indeterminate elements which could be considered to be biasing the sample against juvenile specimens). Diaphyseal cylinders are absent. Digested bones, usually appear as broadly triangular splinters, showing biochemically-abraded surfaces with cupules. Gastric acid attack on compact bone also generates pits derived from the partial dissolution of the cortical surface (Supplementary Fig. S4). This taphonomical alteration associated with carnivore damage is relatively common in these levels (n = 148/9.27% in relation to NR, n = 136/13.85% in relation to NISP by anatomical section); the digestion process can make the remains less identifiable to specific carcass sizes.

In terms of the anatomical distribution of carnivore damage (Supplementary Table S3), pits predominate across all skeletal parts, although scores are more likely to appear on axial elements (with ribs predominating) and long bone diaphyses, whereas epiphyses, cranial fragments, and carpal/tarsal/phalangeal elements, on account of their flat/compacted and cancellous nature, tend to afford a larger proportion of pits. Elements showing a closer ratio of pits:scores ratio (e.g., diaphyseal elements) also show the greatest concentration of notches, plausibly owing to manipulation and consumption of the carcass, involving breakage of long elements in order to gain access to nutritious resources, such as bone marrow.

50 coprolite fragments and disaggregated pellets were retrieved, although only two are >  ~ 25 mm. They may well belong to *Crocuta* from considerations of their size-range, colour, morphology of the best-preserved element (4 × 35 mm; Fig. 2), and archaeological context. Coprolites were also detected in soil micromorphological analyses of these layers^35^. Their future inspection for microspherulites would be useful to confirm their hyaenid nature^39^.

The patterns described here are consistent with previous fieldwork observations at the site. Excavations of the upper Complex 2 levels in 1995 yielded a faunal assemblage similarly dominated by medium- and large-size mammals. In particular, they uncovered a rhinocerotid mandible with a heavily-gnawed ascending ramus and several intact teeth, a rhinocerotid mandibular body and cranium beside which lay three siliceous limestone artefacts, and a large fragment of a proboscidean mandibular body was uncovered nearby; the rhinocerotid teeth had suffered post-depositional damage, probably from falling rocks, and the proboscidean fragment lacked teeth. Elsewhere, an immature proboscidean loph fragment too small for taxonomical identification and the body and neural arch of an immature proboscidean vertebra were found in overlying Complex 1 loose soil (owing to Holocene disturbance), as were the hyaena mandible and also bear teeth that initially were mistaken for hominin incisors until the definitive find in 2019 of an I3 corroborated their ursid designation. Additionally, a bison horn core lying in undisturbed sediment from the upper Complex 2 unit was uncovered when a very large rock was removed.

### Lithic taphonomy

**Figure 6:**
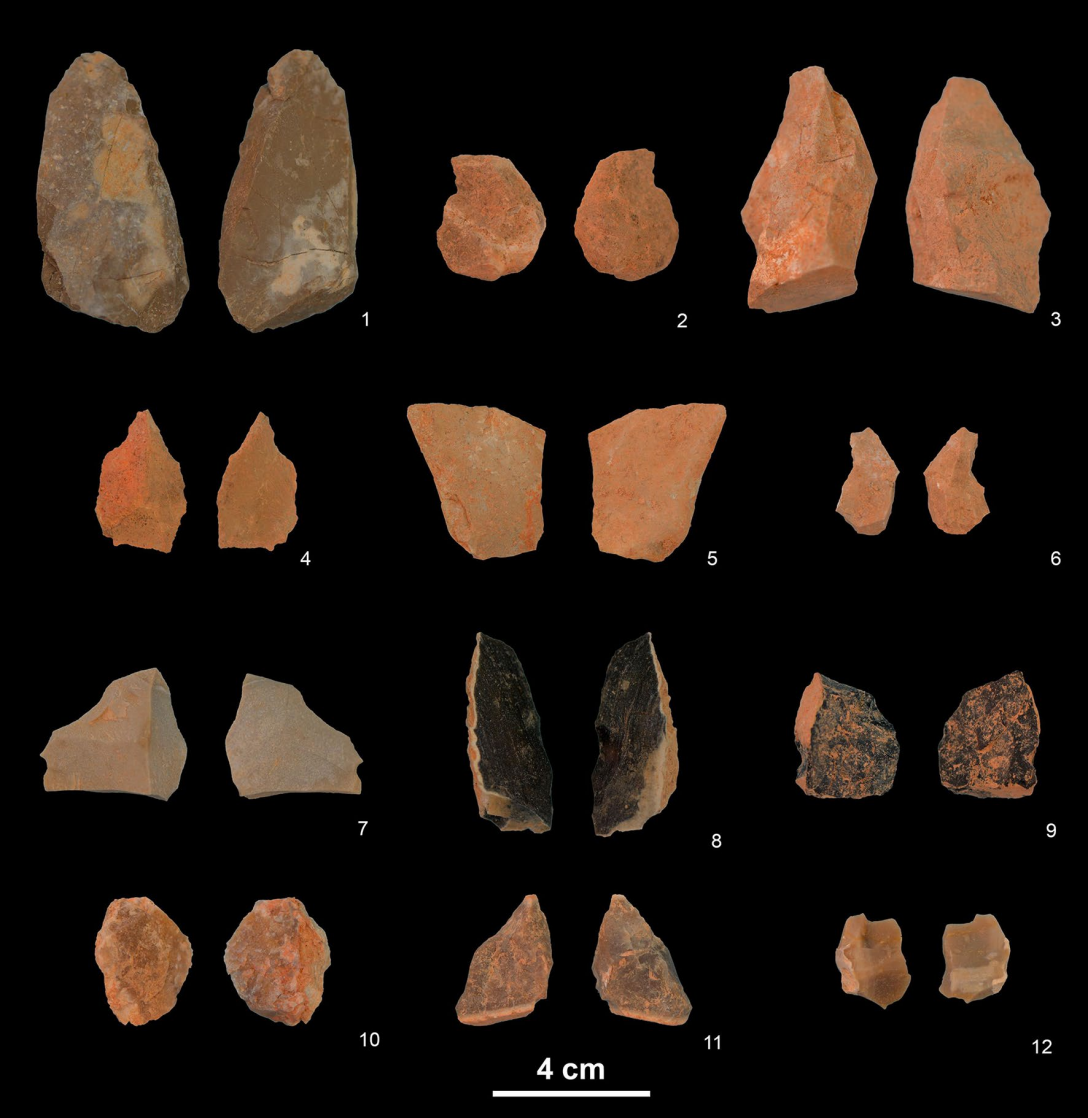
Sample of lithic elements in the CNERQ upper Complex 2 assemblage. 1–6: limestone flakes. 7–12: chert flakes. All lithic implements come from layers 2d–2f, Sectors C1 and C0. Pictures and composition by N.F.R.

Most lithic pieces from layers 2d–2f are of chert, followed by limestone and dolomitic limestone, and sporadically quartzite (Fig. 6; Table 2). These raw materials exist near to the site. Nodules of poor-quality chert exist in a conglomerate outcrop 0.8 km east of the site^40^. Many chert artefacts were fragments or flakes showing incomplete reduction sequences, the frequent retention of cortex indicating a preliminary stage of reduction. Secondary working (simple and abrupt retouch) was infrequent (n = 5) and confined to siliceous pieces. The high proportion of fragments owes to the frangible nature of locally-available, sub-parallelepiped, tabular chert eroded from nearby Jurassic limestone escarpments. Characterised by orthogonal fissure planes, striking it produces flattish, sub-rectangular, laminar fragments far more often than flakes with bulbs of percussion indicative of detachment by conchoidal fracturing. Four hammerstones showing percussive stigmata were among 15 excavated rolled cobbles of limestone. Their mean length L = 78 mm, mean width W = 62 mm, mean thickness T = 49 mm; L/W index = 1.25; T/W index = 1.26. The mean size of flakes and fragments is L = 29 mm, W = 24 mm, T = 11 mm; L/W = 1.20, T/W = 2.18. Mean size of knapping debris: L = 15 mm, W = 11 mm, T = 5 mm. A lone piece showed chemical alteration, in the form of noteworthy patina (perhaps it was taken to the site from elsewhere). Inspection with the binocular field microscope (up to × 40) failed to detect sub-aerial pitting, water-worn smoothing, micro-abrasion, or microfractures.

**Table 2:** Number and type of lithic items, on the basis of raw materials.

Complex 2 layers 2d, 2e, 2f	Limestone	Chert	Quartzite	Other	Total CN (in progress)
Cobbles without percussion marks	10	–	–	1	21
Cobbles with percussion marks	4	–	–	–	13
Cores and core fragments	9	6	2	7	54
Flakes	24	48	2	–	594
Retouched flakes	–	5	–	–	145
Chunks	223	110	7	1	2644
Debris > ** ~ **20 mm	241	120	5	1	235
Debris < ** ~ **20 mm	19	58	–	–	404
TOTALS	191	347	16	349	4110

### Spatial analysis

With regard to the vertical distribution of taphonomical signatures in the assemblage, presence of both carnivore and anthropogenic alterations is seen in all four ~ 100 mm levels (Supplementary Table S4). Anthropogenic modifications are found mostly in layer 2d, both in terms of percussion and cut-marks, although this level also yielded many elements bearing tooth-marks, as well as the greatest concentration of C–D type notches, i.e., overlapping and opposed, respectively, usually ascribed to carnivore activity. The low incidence of cut-marks on bones from layers 2e–f, together with many signs of carnivore damage, such as pits, scores and digested bones, suggests a more restricted anthropogenic input in these levels, with the bulk of this subsample likely owing to hyaenid bone-gathering behaviour.

**Figure 7:**
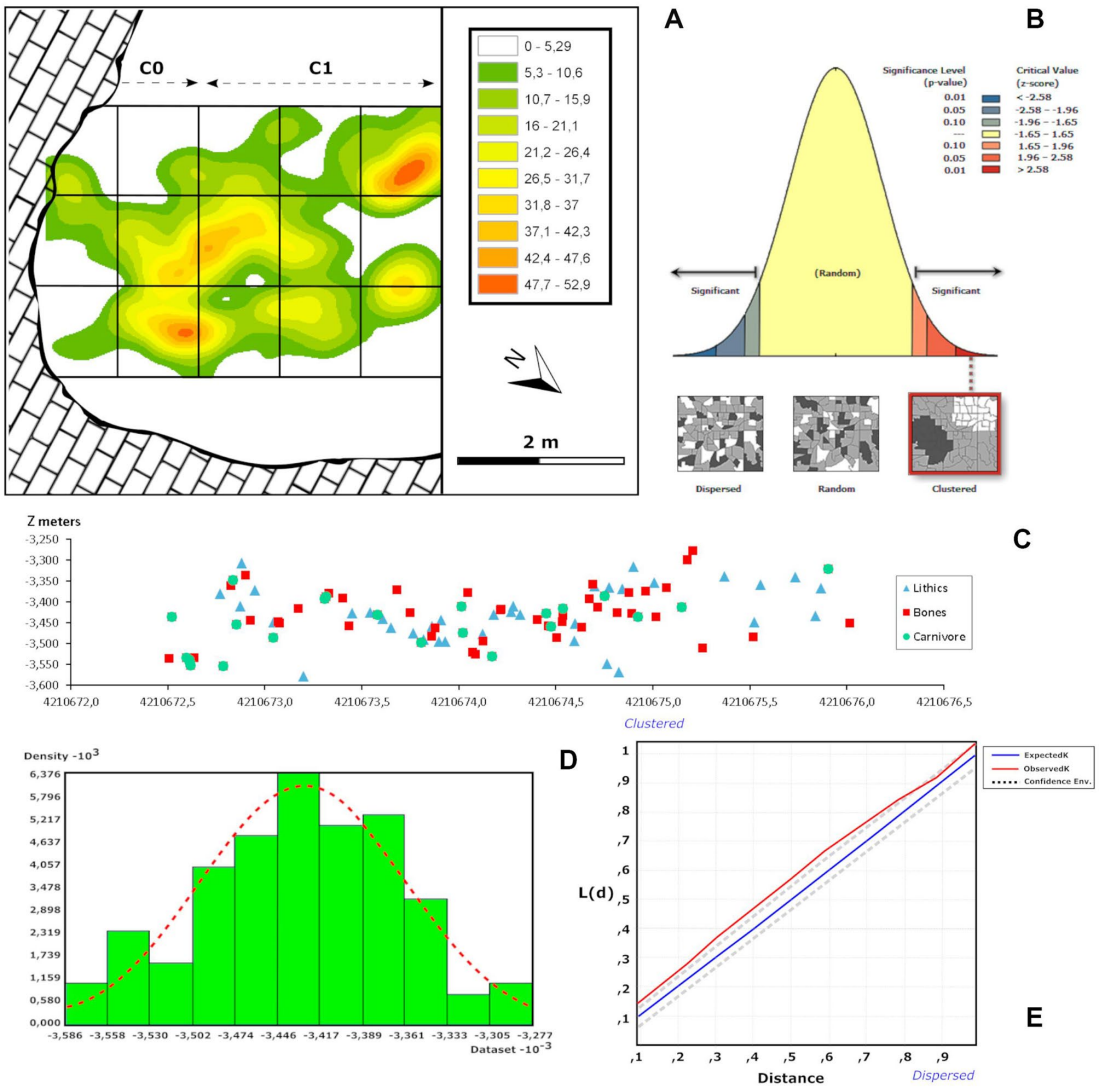
Intrasite spatial analysis for the assemblage of the upper layers of CNERQ Complex 2, carried out using Arcmap’s Spatial Analyst Tools and Geostatistical Analyst Tools. Top (**A**) PCA Kernel density map. (**B**) Moran’s I spatial autocorrelation analysis (clustering, p value < 0.01). (**C**) Vertical distribution plot Unit II layers 2d, 2e, 2f. (**D**) Vertical distribution histogram, showing a normal distribution and a symmetric, unimodal Gaussian curve. (**E**) Ripley’s K Function analysis.

**Figure 8:**
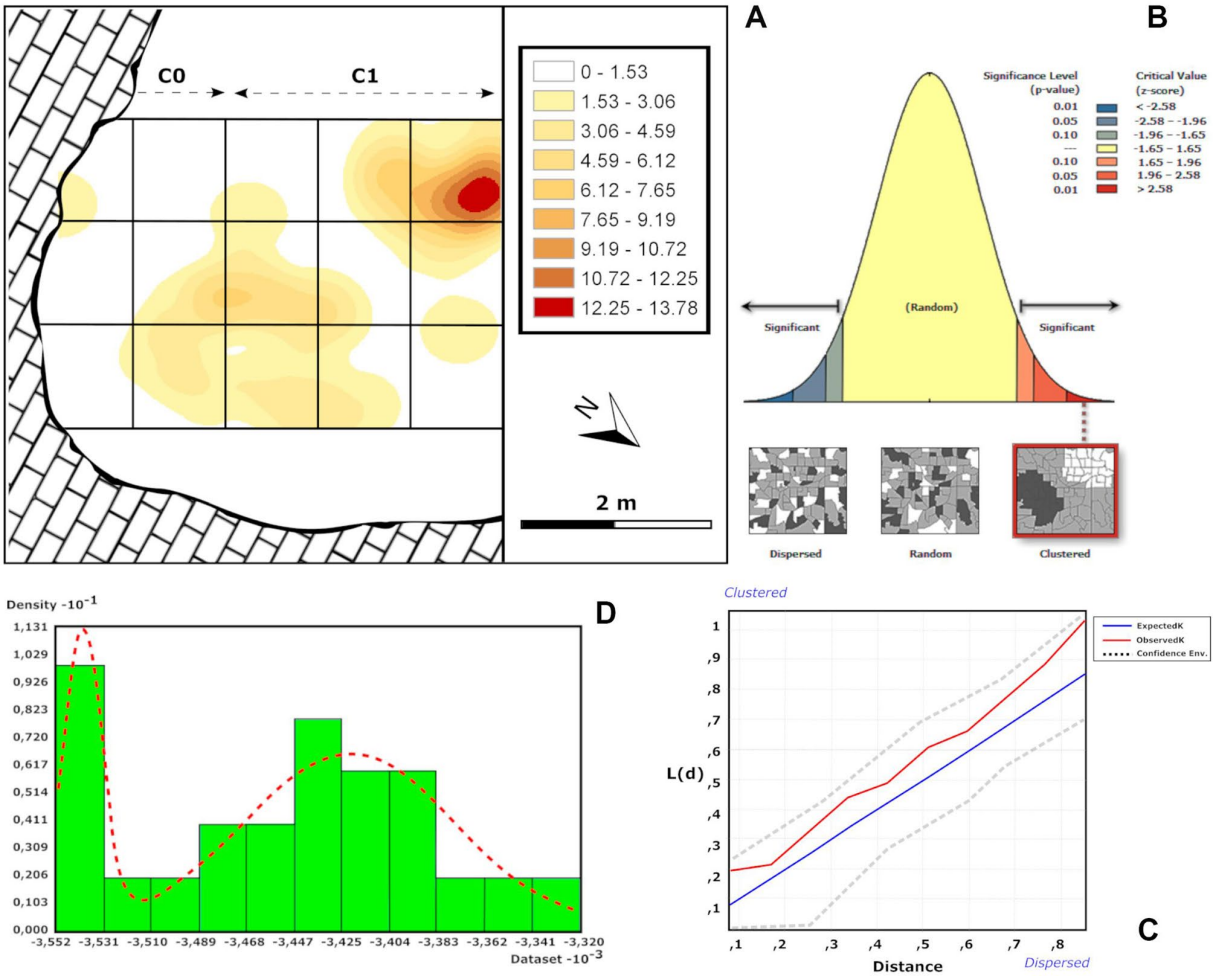
Intrasite spatial analysis for the carnivore-altered faunal assemblage of the upper layers of CNERQ Complex 2, carried out using Arcmap’s Spatial Analyst Tools and Geostatistical Analyst Tools. (**A**) PCA Kernel density map. (**B**) Moran’s I spatial autocorrelation analysis (clustering, p value < 0.01). (**C**) Ripley’s K Function analysis. (**D**) Vertical distribution histogram, showing an asymmetric, bimodal Gaussian curve.

The archaeostratigraphical analyses of the lithic and bone assemblages fail to identify discrete episodes of occupation or frequentation, resulting mostly in a unimodal curve with a statistically normal distribution that need not be taken as corresponding to other than a single statistical “population” represented by the sample analysed (Fig. 7). Nevertheless, a bimodal curve is revealed by the analysis of bones with carnivore damage (Fig. 8), where most values correspond to the intermediate part of the vertical sequence examined here (with a few also from between − 3531 and − 3552 m). It suggests the possibility of an archaeological palimpsest in the upper part of sedimentological Complex 2, between the depths − 3446 and − 3417 m. In contrast, the lower part of Complex 2 registers a series of short, discrete episodes of human activity alternating with short, low-intensity, sedimentary hiatuses, which might correspond to intermittent and recurrent, perhaps seasonal, human use of the site^41^.

The horizontal analyses point towards a pattern with clusters, albeit none that show particular characteristics, whether considered in terms of the size of items recovered, the anatomical parts represented by bones, or the lithic composition (Supplementary Fig. S5). Nevertheless, computations of Moran’s I indicate groupings with statistical significance in all cases (z > 2.58; p < 0.01), hence the probability that the data are distributed randomly is less than 1% (Supplementary Table S5). Moreover, Ripley’s K points to particularly significant clustering when the totality of the finds is analysed, albeit neither when the faunal remains alone are considered, nor yet those with signs of carnivorous impingement (Supplementary Fig. S6; Supplementary Table S6). With regard to the lithic data, it shows a distribution more dispersed over both medium and long distances than that of a purely random distribution, which could owe in part to the quite small number of lithic pieces in the analysis.

**Figure 9:**
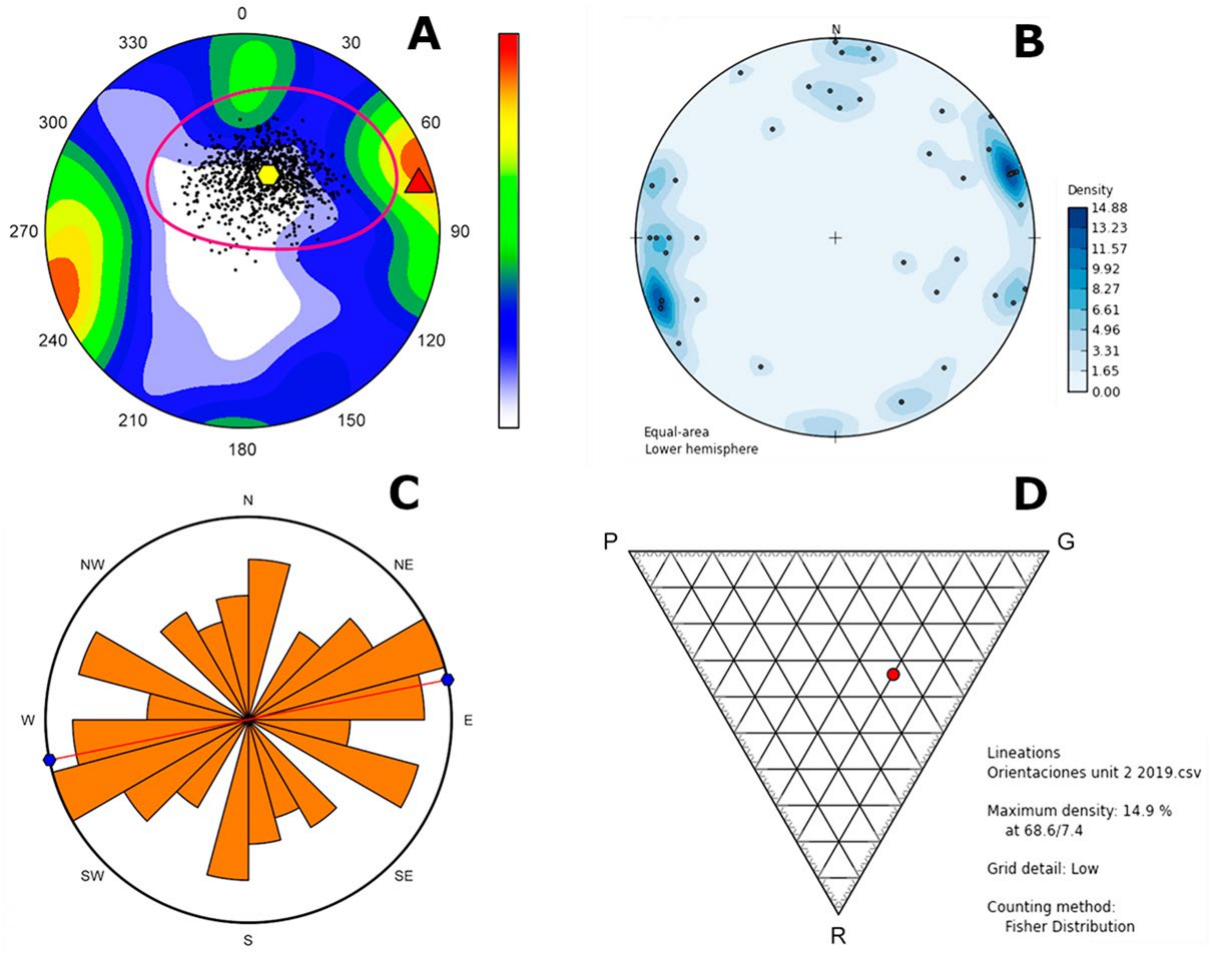
Analyses of archaeological artefacts based on eigenvectors. (**A**) Probability density stereogram, red triangle = maximum eigenvector, yellow hexagon = mean vector, and point cloud is from bootstrapping analysis of 1000 resamples (99.9% confidence). (**B**) Density stereogram with Fisher distribution method. (**C**) Circular histogram of orientations. (**D**) Vollmer PGR plot (performed using orient and OpenStereo softwares; presentation by N.F.R. using Inkscape).

Orientation of artefacts is heterogeneous (Fig. 9, Table 3); they show no overwhelming directionality, notwithstanding an E–W tendency towards maximal density around 68° (Fisher distribution). Values of K = 0.52 and C = 4.80 place the assemblage within a context of planar production, though Benn’s CGI index shows it to lie half-way between planar and linear elaboration (i.e., the planar aspect is not predominant). Application of Vollmer’s PGI index reveals grouped and planar patterns (P = 0.916; G = 0.061; R = 0.021) and low isotropy. Eigenvalues reflect the feeble planar nature of the industry (S1 = S2 ≫ S3).

**Table 3:** Indices calculated for archaeological artefacts based on eigenvectorial analyses.

CN ERQ		V1			V2			V3		
Layer	n	T	Pl	S1	T	Pl	S2	T	Pl	S3
2d–2f	41	75.22	4.50	0.543	344.47	9.4	0.343	190.37	79.5	0.113

Woodcock		Vollmer			Benn				Confidence level	
*K*	*C*	*P*	*G*	*R*	*I*	*E*	*F*	*CGI*		
0.52	4.80	0.916	0.061	0.021	0.208	0.999	0.329	0.465	> 99%	

*V1,* *V2,* *and* *V3* eigenvectors computed for the sample, *T* trend, *B* plunge, *S* eigenvalues, Woodcock: *K* Woodcock’s index K, *C* intensity of preparation, Vollmer: *P* point-cluster index, *G* girdle index, *R* randomness index. Benn: *I* index of isotropy, index of elongation, *F* planar index, *CGI* cluster-girdle index. 99% confidence levels obtained by random bootstrapping.

In summary, the bone assemblage from the upper levels of Complex 2 presents a complex taphonomical history. Several anthropogenic modifications of bone occur, such as percussion and cut-marks, together with several manuports. Traces of carnivore activity also are found, such as juvenile hyaena remains, coprolites, tooth-marks, and digested bones. Assessment of the faunal, lithic, and spatial data suggests that this dual-patterned assemblage appears to represent a palimpsest derived from several, perhaps short-lived, sequences of humans and hyaenid presence, likely with minor inputs from other agents, such as raptors, small mammalian carnivores, and rodents.

## Discussion

The abundant carnivore-related damage documented above reflects the activity of carnivores in determining, or at least altering, the composition of the faunal assemblage. Concurrent with that behaviour were some typically anthropogenic modifications, albeit they appear less frequently. Taphonomical demonstration of the dual-patterned nature of the assemblage leads to reflexion on the formation processes involved, in order to establish the origin and composition of the faunal assemblage.

A fruitful line of research involves assessing the processes by which bones with anthropogenic modifications might have entered the archaeological deposit, i.e., whether they were left-overs, scavenged from another locality in the landscape by bone-gathering carnivores and incorporated into their den assemblages, or whether they represent the remnants of primary anthropogenic use of the cave. If the latter were the case, it then becomes important to assess whether the carnivore damage represents secondary scavenging or is the result of alternative accumulation processes involving other carnivore agents.

A carnivore-ravaged anthropogenic assemblage generally shows considerable alteration and over-representation of fractured splinters^42^. Thus, survival of axial bone and epiphyses should be relatively unlikely, because bone-eating carnivores, such as wolves and hyaenas, tend to select the greasiest and least dense bones first^43,44^. The upper levels of CNERQ Complex 2 contain a highly-fragmented assemblage (87% of specimens <  ~ 30 mm), though a considerable proportion of the fragmentation process must be attributed to post-depositional processes, given high incidence of dry fractures and the relatively low number of bone splinters showing green fractures. Noteworthy also in these levels is the high survival of axial and epiphyseal remains, notwithstanding post-depositional fragmentation that reduced several epiphyses to dense clusters of cancellous bone tissue. Furthermore, no bone with cut-marks exhibited carnivore damage. Therefore, it is unlikely the assemblage from these levels represents exclusively an anthropogenic assemblage with secondary carnivore ravaging.

The main groups of bone-accumulating carnivores in the late Early Pleistocene of southwestern Europe include hyaenids (*Pachycrocuta* *brevirostris* and *Crocuta* *sp.*), and large felids, such as *Panthera* *gombaszoegensis*^4,16,45^. Other large carnivores, such as lions or wolves, mostly consume their prey on the kill spot; even though wolves are capable of modifying bone assemblages, substantial wolf-generated bone accumulations with corresponding modifications have not been recorded^44,46^. The taphonomical signatures of different carnivores can be quite distinct, thereby enabling analytical discrimination when assessing the formation and alteration of bone assemblages. Felids generally leave fewer tooth-marks and inflict fewer fractures on limb bones^47–49^, although modern jaguars (*Panthera* *onca*) have shown some bone-deletion potential in captivity^50^. In contrast, hyenas can consume or chew almost any bony tissue, including antlers^51^. Hyaenas generate den assemblages containing numerous digested bone splinters and coprolites, and leave many tooth marks on bone surfaces^52–54^. Furthermore, hyaenids are social carnivores that exhibit sporadical, scavenging, cursorial predation, and dietary preference for carrion and juvenile or vulnerable prey^55^. In contrast, large felids are usually ambush predators and preferentially target prime-age animals^56^. This ethological distinction is reflected in the attritional skeletal profiles typically found in hyaena dens, in contrast to the “catastrophic” prey profiles left by lions, jaguars, and humans^57^. Nonetheless, actualistic and taphonomical studies show these patterns to be highly variable even at the intraspecific level^58,59^, in relation to the consumption environment, the nature of the site, and the interplay between different individual accumulation agents.

Leopards are solitary and opportunistic hunters which usually protect their caches from other social predators^60–63^. Their accumulations often show a high degree of taxonomical specialisation for mammals of relatively small size, such as Iberian ibex (*Capra* *pyrenaica*) or chamois (*Rupicapra* *rupicapra*), alongside leopard remains. Their assemblages follow typically felid patterns, being dominated by prime-age adults, with high representation of complete limb bones, phalanges, and vertebrae, showing low fragmentation and relatively low tooth-mark counts per specimen^48,49,63^. Such a pattern differs strikingly from the nature of the highly-fragmented bone assemblage and near-absence of vertebrae documented in the upper levels of CNERQ Complex 2, where medium- to large-size animals abound, predominantly cervids. Here, prey selection strategies appear to reflect the main carnivore accumulation agent throughout the Pleistocene, namely, hyaenas^21,64,65^.

Identification of hyaena-accumulated assemblages has relied often on several, highly-contested criteria^52–54^. Whilst no single criterion can be used to determine the nature of a dual-patterned assemblage, the presence of juvenile hyaena remains is widely accepted as circumstantial evidence for in situ existence of a breeding hyaena den^53,54^. Cruz-Uribe^52^ mentioned that carnivore remains are relatively common in hyaena accumulations. Actualistic research has shown that the dens of striped and brown hyaenas tend to have a high proportion of carnivore remains, while spotted hyaenas usually show low carnivore abundance percentages^54^, even in Pleistocene contexts^66^. At CNERQ, the number of carnivore remains excavated in 2019 is very low in both absolute and relative terms, being restricted to an ursid worn lateral incisor (I3) and several juvenile and subadult elements assigned to *Crocuta* sp. These data fit the general pattern described for den assemblages of this genus, despite the degree of intra-specific variability reported in actualistic studies^58^.

Another criterion is the presence of distinctive hyaena damage on bone surfaces, such as the presence of pits, scores, furrowing, and digested bones. The degree of incidence of these depends not only on the age and number of the pups in the breeding den and bone surface preservation, but also on inter-species variability. Brown hyaena assemblages show > 50% of carnivore damage on average, whereas the mean value for both spotted hyaenas and striped hyaenas is below the 50% threshold value proposed by Cruz-Uribe^52^ as signalling a hyaenid accumulation^54,67^. Furthermore, hyaena cubs are often responsible for most bone damage at breeding dens, and their damage is manifested as rather inconspicuous tooth marks^64^, smaller than those generated by adult individuals that are the usual targets of experimental feeding programmes. The matter limits the discriminatory power of traditional bivariate metrical approaches to assess tooth-mark agency, given size similarities between hyaena cubs and smaller carnivores. Regurgitation is a widespread ethological trait of spotted hyaenas^51,68^, even if digested splinters are not always abundant at modern dens^54,59^. The upper levels of Complex 2 at CNERQ provide many instances of carnivore damage (pits, scores, furrowing and digestion) across the bone assemblage (~ 25% of NR), numerous digested splinters, and relatively small tooth marks, together with some larger examples.

Alongside digested splinters, presence of coprolites from localised deposition of droppings in latrines (‘social defaecation’) is another common ethological feature of all extant hyaena species^47,55^. The late Early Pleistocene sites of Cueva Victoria^69^, Atapuerca Trinchera Dolina TD6.1 and La Mina Unit II at Barranc de la Boella have high concentrations of coprolites^39^, whereas others, such as TD6.3^21^ or CNERQ, seem to have lower coprolite counts. This pattern could reflect either a more temporary or episodical use of the CNERQ rock-shelter as a den, which the sporadical presence of anthropogenic elements may imply, or that the location of the main focus of the latrine is to be found outside the excavated surface or even beyond the cave itself.

A diagnostic feature of modern and Pleistocene hyaena accumulations is the existence of bones retaining almost complete diaphyses while exhibiting gnawed epiphyses (bone "cylinders"). Modern spotted hyaena dens have yielded up to 22% of bone cylinders in high-competition environments^54^. However, bone cylinders are uncommon in the hyaena den assemblage of Atapuerca TD6.3^21^, and they are also seemingly absent from CNERQ. Perhaps this pattern is exclusively resulting from post-depositional fragmentation, given the high proportion of dry fractures. Nonetheless, different frequencies of these alterations are related also to competition for carcass consumption or hunger levels in wild hyaenas^59^. As such, another plausible and complementary conjecture is that perhaps the social hunting prowess of *Crocuta* sp.^12,55^ allows them to rely less on bone nutrients in periods of low competition, such as the latest Early Pleistocene in Western Europe^70^. In that regard, the behaviourally-flexible *Crocuta* might have differed from the larger and more scavenging-reliant *Pachycrocuta* *brevirostris*, shown to have modified heavily the bones of even megafaunal species present in their accumulations^23,47,69,71^.

Cursorial predation by hyaenas is regarded as giving rise to attritional age profiles in their bone assemblages, because of their tendency to prey on vulnerable individuals^55,57,68^. Attritional profiles are characterized by many individuals in the youngest age class, few individuals in the middle-age "prime adult” class, and relatively more individuals in the senile age class^52^. This last age class is not always readily detectable in archaeological assemblages given the need for a representative dental sample. Even though juvenile elements (n = 90) represent only 5.6% of the assemblage, 30% of tooth-marks (n = 80/274) were found on these juvenile elements. Furthermore, no juvenile element showed anthropogenic modifications, so it would appear that bone-gathering carnivores were primarily responsible for the presence of juvenile specimens at the site, corroborating the inference that indeed they did prey on vulnerable individuals. This pattern would be consistent with the hyaenid attribution of carnivore agency at the dual-patterned upper levels of CNERQ Complex 2, even if no attritional signature appears in the skeletal profile overall. The absence of a generally attritional skeletal profile was recorded also in Atapuerca TD6.3^21^, although the heterogeneous nature of the layers considered in the taphonomical study of the TD6.3 assemblage could have biased the quantification overall^72^; similarly, we argue that contributions from other agents and post-depositional fragmentation are likely to have contributed to this variability, rather than that it necessarily represents a higher incidence of secondary scavenging at these late Early Pleistocene sites. Spotted hyaena den assemblages are particularly prone to the potential erasure of the compositional integrity of their skeletal profiles by other biological and physico-chemical agents, because this species usually consumes its prey where it is killed^55^ and appears to have lower rates of bone accumulation per year than are found among other extant hyaena species^73^.

Other criteria are the correlation between ungulate size and the cranial-postcranial ratio, and the under-representation in hyaena accumulations of small, hard bones other than teeth (e.g., carpals, tarsals, and phalangeal elements)^52,67^. Spotted hyaenas, however, have been shown to bring cranial remains of (juvenile) large ungulates to their dens^53,54^. Moreover, “small, hard bones are only consistently under-represented in the dens of striped hyenas”^54^; therefore, behavioural variability also seems to play an important role. Cranial remains are relatively common in the CNERQ upper Complex 2 assemblage, whilst carpals, tarsals, and phalanges are present in modest proportions (1.5%). The latter also show a high incidence of digestion-related damage on their cortical surfaces (Fig. S3, Table S1), suggesting that consumption patterns of hyaenas could have given rise to under-representation of these elements at the site, given that small foot bones are represented frequently in the regurgitations of spotted hyaenas^53^. The taphonomy of antler remains is complex, because some antlers undergo pitting in vivo during a cervid’s lifetime, rodents can cause gnawing damage on antler remains, and a high proportion of horns and antlers is no longer considered indicative of hyaena accumulations^53,57^. Nonetheless, the presence of several *Megaloceros* antlers with some evidence of carnivore ravaging in the CNERQ upper Complex 2 assemblage (Fig. 3) would be consistent with the expected behavioural patterns of *Crocuta* sp. and would further support the role of a large carnivore in the generation and/or modification of the bone assemblage.

Regarding the human input to the dual-patterned nature of the bone assemblage, anthropogenic modification appears less frequently in the assemblage than carnivore damage. Nonetheless, cut-marks are seen mainly on the central diaphyses and axial remains of large- and medium-sized carcasses, a pattern generally associated with primary access to carcasses^74^. These bones with anthropogenic traces lack typical carnivore damage, such as pits, scores, furrowing, or digestion. Furthermore, no juvenile elements show anthropogenic modifications, notwithstanding a high incidence of carnivore tooth marks on them. Notches are relatively uncommon (2.77% on long-bone diaphyses); their proportions and nature mostly demonstrate a combination of double-overlapping and double opposing notches [Types C-D] with some presence of single ones [A and B], mirroring the data for the Lake Eyasi modern hyaena den^59^. Some notches (particularly those of types A and B) might derive from anthropogenic bone fragmentation. This pattern suggests differential access to carcasses by early humans and hyaenas, rather than a reliance of hyaenas on scavenged remains from human hunters or vice-versa.

The lithic assemblage from the upper levels of CNERQ Complex 2 suggests brief periods of occupation or frequentation. Lithic analysis demonstrates a somewhat nondescript assemblage affected by heavily-skewed reduction sequences and local availability of raw materials, especially poor-quality chert and limestone cobbles. The proportion of such cobbles (manuports) is considerably higher in the sample excavated in the uppermost layers than is the case for deeper sediments at the site. Perhaps they represent a collection made for a particular purpose and left as a cache for further use^75^. Fluviatile processes hardly can have been responsible for depositing them, given that sedimentary micromorphology is incompatible with high transport energy^35^. As already remarked, rolled gravel between 5 and 50 mm in size is scarce throughout the Cueva Negra sedimentary sequence, thereby highlighting a granulometrical discontinuity that is atypical of fluviatile aggradations of river gravels. Several excavated water-worn cobbles > 50 mm in size have shapes uncharacteristic of heavy river gravel, lack traces of abrasion, chemical alteration, or patination, and often found broken or flaked: most seem to be Palaeolithic manuports of chert, quartzite and hard siliceous limestone that were extracted from an Upper Miocene marine conglomerate outcrop 0.8 km east of the cave^40^.

The upper layers in Complex 2 contain evidence of various brief episodes of human occupation or frequentation and at least two when hyaenas were present, with remains interposed as in a palimpsest, albeit not always separable, owing probably to post-depositional carnivore bioturbation, and fluviatile or other impingements that contributed to formation of a bed in which vertical alteration is more pronounced than is detectable horizontally. The presence of rodent gnawing damage on dry and weathered bones renders plausible that some of the bone fragments were introduced by these agents, compounding further the notion of an archaeological palimpsest. Spatial analyses reflect some patterns of clustering or grouping, and the planar distribution of lithic elements, though no pattern is significant enough for inference of either dispersal or important alteration having taken place, therefore suggesting only minor redistribution and displacement of remains. The lengthwise dip of long-bone fragments and other items indicates that they were deposited on an uneven surface. It reflects the influence of fluctuations in the water level of the erstwhile swampy lake fed by the river, which nevertheless do not seem to have produced significant displacement of the remains.

## Conclusions

Both taphonomical and taxonomical variables support existence of hyaenid occupations in the upper levels of CNERQ Complex 2. The abundance of dry fractures and the presence of weathering and rodent gnawing in these levels are in stark contrast to the pattern found in other sedimentary units^76^. Slower sedimentation rates, alongside some degree of bioturbation detected at the micromorphological level^35^ compatible with the burrowing activities of hyaenas in their dens, likely favoured the generation of an archaeological palimpsest. The identification of human and carnivore occupations in the same space is a common occurrence in karst contexts^77^. The Cueva Negra data has relevant implications for understanding the biogeographical configuration of the late Early Pleistocene landscapes of southern Europe.

Dispersal of *Crocuta* into Europe from Africa and Asia took place at the end of the Villafranchian (after the Jaramillo normal polarity sub-chron), during MIS 21–MIS 19, as documented at Atapuerca-Trinchera Dolina from TD4/5 onwards^21,78^ and at Cueva Negra del Estrecho del Río Quípar^28^. *Crocuta* dispersal in the late Early Pleistocene coincided with a more consolidated presence of early humans in southern Europe^28,74^, and the faunal turnover during the Early-to-Middle Pleistocene transition from 41 ka climatic cycles to cyclical glacial periodicity of 100 ka^12^. This spread of social hunters, such as *Crocuta* and *Homo*, also corresponded with the decline of late Villafranchian solitary hypercarnivores and large scavengers^6^, such as the sabre-toothed *Megantereon* *whitei,* the Pleistocene Eurasian jaguar (*Panthera* *gombaszoegensis*), and the large hyaenid *Pachycrocuta* *brevirostris*, which are well represented at the 0.9–1.0 Ma site of Cueva Victoria^69^.

Presence of early humans, hyaenas, and other predators in the Quípar valley was surely influenced by accessibility to water, shelter, and raw materials. Nonetheless, the extent of their coexistence must have been determined by their density within the landscape, the temporality of their hunting and breeding seasons, and the shifting quality and availability of animal and plant resources^79^. Evidence of human presence, given primarily by Palaeolithic artefacts and anthropogenic modifications on faunal remains, is consistent with recurrent frequentation of the CNERQ rock-shelter by early humans. The hyaena breeding den appears to have existed only during a limited period of time in relation to the overall sedimentary sequence, although hyaenas played a substantial part in the configuration of the faunal assemblage from the upper levels of Complex 2. Plausibly, human absences no doubt favoured use of the site as a breeding den by hyaenas, or uses by other carnivores (e.g., hibernating bears). Moreover, hyaenas and humans likely were in quasi-simultaneous coexistence with other small predators, which were responsible for the accumulation of the micromammal, avian, and herpetological elements. A similar pattern of differential intensity of occupation characterises the transition between TD6.2 and TD6.3 at Atapuerca, as well as within TD6.3 itself^21,72^. The co-existence of carnivores and humans and their relative density within the landscape seems to have influenced the distribution of carnivore dens and the settlement dynamics of latest Early Pleistocene humans in South-Western Europe. It appears likely that early human “risk-management” strategies regarding the use of rock-shelters and other habitat sites favoured avoiding confrontations with fierce carnivores.

## Methods

The following data relate to the excavation in 2019 of 15 m^2^ of the uppermost layers of the Pleistocene sedimentary deposit, which correspond to the artificial subunits 2d, 2e and 2f of Complex 2 in the 3 × 3 m sectors C1 and C0 at the rear of the rock-shelter. For manual excavation, the aforementioned 3 × 3 m sectors were partitioned into 1 × 1 m squares. Each was subdivided into 0.5 × 0.5 m areas that were excavated in artificial spits no more than ~ 50 mm deep (2c_i_, 2c_ii_, 2d_i_, 2d_ii_, etc.), or less if changes were detected in the geoarchaeological characteristics of the sediment. In that way, we constrained the spatial coordinates of those finds that had escaped precise georeferencing with the total station, as we aimed to record every archaeological object >  ~ 20 mm in length uncovered during excavation. All excavated sediment was washed over nests of 3 stainless-steel sieves with meshes of 8, 4 and 2 mm, respectively, thereby ensuring recovery of such tiny items as rodent teeth and minute chips of knapped chert.

The decision to encompass in this study only the aforementioned artificial sublevels of the upper Complex 2 is a conscious attempt to prevent any kind of potential mixing from actual micro-archaeostratigraphical subdivisions, given the minor differential sedimentary input at the base of 2f—which may create a non-geologically but perhaps archaeologically-significant difference in the taphonomical history of the site. This minor differential sedimentary input is recorded in the geoarchaeological sequence of the site^35^ (Fig. 1).

### Bone taphonomy

Taxonomical identifications were made following standard protocols, with the aid of reference collections and anatomical atlases. Those elements for which taxonomical identification was not possible were classified on the basis of animal weight-size: our category “small” corresponds to Bunn’s levels 1–2; medium corresponds to Bunn’s level 3a; “large” corresponds to Bunn’s categories 3b and 4, and “very large” encompasses Bunn’s categories 5–6^80^. Faunal remains were quantified on the basis of the number of remains (NR), the number of identifiable specimens (NISP), and the minimum number of individuals (MNI), taking into account bone laterality and ontogenetic age for the estimation of the latter^81,82^. Age was estimated on the basis of dental ontogeny and cortical bone porosity.

Taphonomical research was carried out on all macrofaunal bone specimens >  ~ 10 mm, using hand-held 10 ×–20 × lenses, as proposed by Blumenschine^83^. The identification of the BSM (bone surface modification) was undertaken following established criteria^80,84,85^. Cut-mark quantification has been made on the basis of individual cut-marks on bone fragments that show a good cortical preservation. Regarding tooth-marks and percussion marks, we have followed the methods outlined by Blumenschine^83^. For the characterisation of notches, we followed Capaldo and Blumenschine^86^. Carnivore ravaging was assessed also by establishing the following damage patterns: pits, scores, punctures, furrowing, crenulated edges, licking, pitting, and digestion, as outlined in Saladié et al.^21^. Bone fragmentation was categorised on the basis of shaft circumference and the length and width of the remains in order to appraise the nature and intensity of fragmentation^80,87^. The almost entire absence of semi-complete long bones precluded any assessments of carnivore agency on the basis of “taphotype” markers^88^.

For the analysis of skeletal part profiles, we classified remains into cranial (horn-antler, cranium, maxilla, mandible), axial (vertebra, rib, pelvis, scapula), compact bones (carpals/tarsals, sesamoids, and phalanges), and subdividing non-identifiable limb bones into epiphyses and diaphyses^89,90^. Unclassifiable elements were left as indeterminate. Furthermore, we also recorded post-depositional alterations in order better to understand and reconstruct site formation processes, assess site integrity, and evaluate the role of different biotic agents in the accumulation of the faunal assemblage. We documented and quantified the presence and stage of manganese oxide staining (0–3; Table S8)^91^, weathering (0–2+, after Behrensmeyer^92^), biochemical alterations (0–2), the incidence of mechanical alterations, such as trampling^93^ (Table S9), the presence of rodent gnawing, and the formation of authigenic minerals on bone surfaces.

Photographs were taken using a *Celestron* *Pro* *Digital* *Microscope* (0–200×, 5MP) and a 40 MP *Leica* camera, and processed using *Helicon* *Focus* and *Adobe* *Photoshop* software.

### Lithic taphonomy

The taphonomical study of bone remains is complemented here by considerations of the lithic finds and spatial analyses of horizontal and vertical patterns of distribution with particular reference to the multidimensionality of the archaeological space and the concept of archaeological palimpsests^94^.

Lithic pieces were analysed both from the 2019 excavation in sectors C1 and C0 of layers 2d, 2e and 2f, and also from earlier excavations in the uppermost sediments of sectors C2, C3 and B3. Analytical criteria used here are informed by well-known proposals concerning reduction sequences of raw materials^95^. Once sorted into different types of raw materials, pieces were classified from the standpoints of the kinds of primary base, stage of reduction, size, and the extent of physico-chemical alteration and abrasion—both are of particular taphonomical interest in fluviatile environments^96^. Moreover, it should be borne in mind that size-ranges can be affected by sedimentary processes^97,98^. Also, they may be skewed were water entering different parts of a site to have affected them differentially owing to spatio-temporal variations in its transport-energy. Skewing may result from post-depositional processes that can cause vertical displacement of buried objects^99,100^. Of course, some degree of skewing can owe, as well, to the limited spaces available within which recovery and sampling of finds take (or can take) place at a site.

### Spatial analysis

Spatial analysis was performed on lithic and bone items from the 15 m^2^ excavated in C1 and C0. *Arcgis*, *Orient* y *OpenStereo* softwares were used for statistical computation and graphical representation.

Analysis of vertical distributions is displayed in graphs of dispersal and histograms of probability frequencies and Gaussian distribution, following determination of sample normality^41^. Different scales of reference are employed: namely, all finds with coordinates defined by georeferencing; all bone items; and all lithic items together with bone fragments showing signs of carnivorous impingement.

Analysis of horizontal distributions is obtained from the projection of points in Kernel density analysis, taking into account: the total sum of all items; all bone pieces; bone fragments showing signs of carnivorous impingement; identifiable anatomical parts; distribution by size-range; and all lithic pieces and manuports.

For all the aforementioned variables, analysis of spatial correlation was carried out using Moran’s I based on the (x, y) data for each item, thereby enabling evaluation of the degrees of randomness, dispersal, or assemblage grouping. Likewise, Ripley’s K function in multi-distance spatial cluster analysis was used to measure the type, intensity, and range of spatial patterning, with maximal reliability for the results being attained by Monte Carlo simulation with 999 permutations and correction for the edge-effect that can distort calculation of Ripley’s K and is a consequence of the existence of the physical boundary or edge that surrounds and delimits the excavated surface^101^.

A compass and clinometer were used to define the orientation and dip of items uncovered, thereby permitting statistical analysis of eigenvectors and assessment of post-depositional or other sedimentary disturbance to the archaeological assemblage^97,102,103^. Azimuth data were determined for those archaeological artefacts > ~ 30 mm in size (and ecofacts > ~ 100 mm) that were uncovered revealing a major axis giving a length/width index > 1.5 (n = 41). Analysis of eigenvectors, eigenvalues, K and C ratios, and Vollmer’s Index were computed and are given as circular histograms, stereograms, and Vollmer diagrams (random bootstrapping provides values with 99% confidence).

## Supplementary Information

Supplementary Information.

## Data Availability

Any relevant taphonomic, spatial and/or lithic data not included in the present paper or Supplementary Information will be made available upon request.

## References

[1] Brugal JP, Fosse P (2004). Carnivores et hommes au Quaternaire en Europe de l`Ouest. Rev. Paléobiol..

[2] Rodríguez-Gómez G (2016). On the ecological context of the earliest human settlements in Europe: Resource availability and competition intensity in the carnivore guild of Barranco León-D and Fuente Nueva-3 (Orce, Baza Basin, SE Spain). Quatern. Sci. Rev..

[3] Blasco R (2011). Hiding to eat: The role of carnivores in the early Middle Pleistocene from the TD8 level of Gran Dolina (Sierra de Atapuerca, Burgos, Spain). J. Archaeol. Sci..

[4] Pineda A (2015). Coexistence among large predators during the Lower Paleolithic at the site of La Mina (Barranc de la Boella, Tarragona, Spain). Quatern. Int..

[5] Van der Made J (2011). Biogeography and climatic change as a context to human dispersal out of Africa and within Eurasia. Quatern. Sci. Rev..

[6] Palombo MR (2008). Carnivora dispersal in Western Mediterranean during the last 2.6 Ma. Quatern. Int..

[7] Arzarello M, Peretto C, Moncel M-H (2015). The Pirro Nord site (Apricena, Fg, Southern Italy) in the context of the first European peopling: Convergences and divergences. Quatern. Int..

[8] Mosquera M, Ollé A, Rodríguez X-P (2013). From Atapuerca to Europe: Tracing the earliest peopling of Europe. Quatern. Int..

[9] Agustí J (2015). Chronological and environmental context of the first hominin dispersal into Western Europe: The case of Barranco León (Guadix-Baza Basin, SE Spain). J. Hum. Evol..

[10] Despriée J (2010). Lower and middle Pleistocene human settlements in the Middle Loire River Basin, Centre Region, France. Quatern. Int..

[11] Cauche D (2009). Les stratégies de débitage dans les industries lithiques archaïques des premiers habitants de l’Europe. Anthropologie.

[12] Sardella R, Petrucci M (2012). The earliest Middle Pleistocene *Crocuta**crocuta* (Erxleben, 1777) at Casal Selce (Rome, Italy). Quatern. Int..

[13] Walker MJ (2017). Palaeolithic Pioneers: Behaviour, Abilities, and Activity of Early Homo in European Landscapes Around the Western Mediterranean Basin ~1.3 to 0.05 Ma.

[14] Carrión JS, Walker MJ (2019). Background to Neanderthal presence in Western Mediterranean Europe. Quatern. Sci. Rev..

[15] Duval M (2018). The first direct ESR dating of a hominin tooth from Atapuerca Gran Dolina TD-6 (Spain) supports the antiquity of *Homo**antecessor*. Quat. Geochronol..

[16] Turner A, Antón M (1996). The giant hyaena *Pachycrocuta**brevirostris* (Mammalia, Carnivora, Hyaenidae). Geobios.

[17] Arribas A, Palmqvist P (1999). On the ecological connection between Sabre-tooths and Hominids: Faunal dispersal events in the Lower Pleistocene and a review of the evidence for the first human arrival in Europe. J. Archaeol. Sci..

[18] Madurell-Malapeira J (2017). Were large carnivorans and great climatic shifts limiting factors for hominin dispersals? Evidence of the activity of *Pachycrocuta**brevirostris* during the Mid-Pleistocene Revolution in the Vallparadís Section (Vallès-Penedès Basin, Iberian Peninsula). Quatern. Int..

[19] Bourguignon L (2016). Bois-de-Riquet (Lézignan-la-Cèbe, Hérault): A late Early Pleistocene archeological occurrence in southern France. Quatern. Int..

[20] Espigares MP (2013). *Homo* vs *Pachycrocuta*: Earliest evidence of competition for an elephant carcass between scavengers at Fuente Nueva-3 (Orce, Spain). Quatern. Int..

[21] Saladié P (2019). The TD6.3 faunal assemblage of the Gran Dolina site (Atapuerca, Spain): A late Early Pleistocene hyena den. Hist. Biol..

[22] Saladié P (2014). The role of carnivores and their relationship to hominin settlements in the TD6.2 level from Gran Dolina (Sierra de Atapuerca, Spain). Quatern. Sci. Rev..

[23] Madurell-Malapeira J, Pérez-García A, Gascó F, Gasulla JM, Escaso F (2011). Taphonomic approach to the last Villafranchian faunas of Europe: The layer 7 of the Vallparadís Estació local section (Vallès-Penedès Basin, NE Iberian Peninsula). Viajando a Mundos Pretéritos.

[24] Faith JT, Behrensmeyer AK (2006). Changing patterns of carnivore modification in a landscape bone assemblage, Amboseli Park, Kenya. J. Archaeol. Sci..

[25] Binford LR (1981). Bones. Ancient Men and Modern Myths.

[26] Walker MJ (2013). Cueva Negra del Estrecho del Río Quípar (Murcia, Spain): A late Early Pleistocene hominin site with an “Acheulo-Levalloiso-Mousteroid” Palaeolithic assemblage. Quatern. Int..

[27] Walker MJ (2016). A view from a cave: Cueva Negra del Estrecho del Río Quípar (Caravaca de la Cruz, Murcia, southeastern Spain). Reflections on fire, technological diversity, environmental exploitation, and palaeoanthropological approaches. Hum. Evol..

[28] Walker MJ (2020). Cueva Negra del Estrecho del Río Quípar: A Dated late early Pleistocene Palaeolithic Site in Southeastern Spain. J. Paleo Arch..

[29] Scott G, Gibert L (2009). The oldest hand axes in Europe. Nature.

[30] López-Jiménez A (2020). Small-mammal indicators of biochronology at Cueva Negra del Estrecho del Río Quípar (Caravaca de la Cruz, Murcia, SE Spain). Hist. Biol..

[31] Walker MJ (2019). New chronological constraints for the Lower Palaeolithic site of Cueva Negra del Estrecho del Río Quípar, Caravaca de la Cruz, Murcia, Spain: Preliminary ESR dating of the late Early Pleistocene fauna. Proc. Eur. Soc. Stud. Hum. Evol..

[32] Carrión JS (2003). Glacial refugia of temperate, Mediterranean and Ibero-North African flora in south-eastern Spain: New evidence from cave pollen at two Neanderthal man sites. Glob. Ecol. Biogeogr..

[33] Walker MJ (2016). Combustion at the late Early Pleistocene site of Cueva Negra del Estrecho del Río Quípar (Murcia, Spain). Antiquity.

[34] Rhodes SE (2016). Fire in the Early Palaeolithic: Evidence of small mammal burning at Cueva Negra del Estrecho del Río Quípar, Murcia, Spain. J. Archaeol. Sci. Rep..

[35] Angelucci DE (2013). Rethinking stratigraphy and site formation of the Pleistocene deposit at Cueva Negra del Estrecho del Quípar (Caravaca de la Cruz, Spain). Quatern. Sci. Rev..

[36] Linares-Matás GJ (2019). A geometric-morphometric assessment of three-dimensional models of experimental cut-marks using flint and quartzite flakes and handaxes. Quatern. Int..

[37] López-Cisneros P (2019). Applying new technologies to the taphonomic study of La Lluera (Asturias, Spain). Geometric morphometrics and the study of bone surface modifications (BSM). Quatern. Int..

[38] Maté-González MÁ (2019). New technologies applied to modelling taphonomic alterations. Quatern. Int..

[39] Pineda A (2017). Characterizing hyena coprolites from two latrines of the Iberian Peninsula during the Early Pleistocene: Gran Dolina (Sierra de Atapuerca, Burgos) and la Mina (Barranc de la Boella, Tarragona). Palaeogeogr. Palaeocl..

[40] Zack W (2013). Stone procurement and transport at the late Early Pleistocene site of Cueva Negra del Estrecho del Río Quípar (Murcia, SE Spain). Quartär.

[41] Fernández Ruiz N (2018). Cueva Negra del Estrecho del Río Quípar (Caravaca de la Cruz, Murcia, SE Spain): Intrasite analysis of a late Early Pleistocene Palaeolithic palimpsest. Proc. Eur. Soc. Study. Hum. Evol..

[42] Binford LR (1988). Hyena scavenging behavior and its implications for the interpretation of fauna1 assemblages from FLK 22 (the Zinj Floor) at Olduvai Gorge. J. Anthropol. Archaeol..

[43] Marean CW, Spencer LM (1991). Impact of carnivore ravaging on zooarchaeological measures of element abundance. Am. Antiquity.

[44] Yravedra J (2011). A taphonomic study of wild wolf (*Canis**lupus*) modification of horse bones in Northwestern Spain. J. Taphonomy.

[45] Palmqvist P (2011). The giant hyena *Pachycrocuta**brevirostris*: Modelling the bone-cracking behavior of an extinct carnivore. Quatern. Int..

[46] Mech LD (1970). The Wolf: The Ecology and Behavior of an Endangered Species.

[47] Brain CK (1981). The Hunters of the Hunted? An Introduction to African Cave Taphonomy.

[48] Parkinson JA (2015). Characterizing felid tooth marking and gross bone damage patterns using GIS image analysis: An experimental feeding study with large felids. J. Hum. Evol..

[49] Arriaza MC (2019). Characterising leopard as taphonomic agent through the use of micro-photogrammetric reconstruction of tooth marks and pit to score ratio. Hist. Biol..

[50] Rodríguez-Alba JJ (2019). First assessments of the taphonomic behaviour of jaguar (*Panthera**onca*). Quatern. Int..

[51] Sutcliffe AJ (1970). Spotted hyaena: Crusher, gnawer, digester and collector of bones. Nature.

[52] Cruz-Uribe K (1991). Distinguishing hyena from hominid bone accumulations. J. Field Archaeol..

[53] Pickering TR (2002). Reconsideration of criteria for differentiating faunal assemblages accumulated by hyenas and hominids. Int. J. Osteoarchaeol..

[54] Kuhn B (2010). Examining criteria for identifying and differentiating fossil faunal assemblages accumulated by hyenas and hominins using extant hyenid accumulations. Int. J. Osteoarchaeol..

[55] Kruuk H (1972). The Spotted Hyaena: A Study of Predation and Social Behavior.

[56] Gidna AO (2014). An ecological neo-taphonomic study of carcass consumption by lions in Tarangire National Park (Tanzania) and its relevance for human evolutionary biology. Quatern. Int..

[57] Stiner MC (1991). Food procurement and transport by human and nonhuman predators. J. Archaeol. Sci..

[58] Lam YM (1992). Variability in the behaviour of spotted hyaenas as taphonomic agents. J. Archaeol. Sci..

[59] Prendergast ME, Domínguez-Rodrigo M (2008). Taphonomic analyses of a hyena den and a natural-death assemblage near lake Eyasi (Tanzania). J. Taphonomy.

[60] Yravedra J (2006). Acumulaciones biológicas en yacimientos arqueológicos: Amalda VII y Esquilleu III–IV. Trabajos Prehist..

[61] Yravedra J (2010). A taphonomic perspective on the origins of the faunal remains from Amalda Cave (Spain). J. Taphonomy.

[62] de Ruiter DJ, Berger LR (2000). Leopards as taphonomic agents in dolomitic caves implications for bone accumulations in the hominid-bearing deposits of South Africa. J. Archaeol. Sci..

[63] Sauqué V, Sanchís A (2017). Leopards as taphonomic agents in the Iberian Pleistocene, the case of Racó del Duc (Valencia, Spain). Palaeogeogr. Palaeocl..

[64] Fourvel J-B (2012). Consumption of ungulate long bones by pleistocene hyaenas: A comparative study. J. Taphonomy.

[65] Crezzini J (2016). A spotted hyaena den in the Middle Palaeolithic of Grotta Paglicci (Gargano promontory, Apulia, Southern Italy). Archaeol. Anthrop. Sci..

[66] Sala MTN (2012). Taphonomic study of the Búho and Zarzamora caves. Hyenas and humans in the Iberian Plateau (Segovia, Spain) during the Late Pleistocene. J. Taphonomy.

[67] Stewart M, Andrieux E, Clark-Wilson R (2021). Taphonomy of an excavated striped hyena (*Hyaena**hyaena*) den in Arabia: Implications for paleoecology and prehistory. Archaeol. Anthropol. Sci..

[68] Egeland AG (2008). Taphonomic analysis of a modern spotted hyena (*Crocuta**crocuta*) den from Nairobi, Kenya. J. Taphonomy.

[69] Gibert L, Ferràndez-Cañadell C (2015). Geología y paleontología de Cueva Victoria. Mastia.

[70] Rodríguez J, Mateos A (2018). Carrying capacity, carnivoran richness and hominin survival in Europe. J. Hum. Evol..

[71] Arribas A, Palmqvist P (1998). Taphonomy and paleoecology of an assemblage of large mammals: Hyaenid activity in the Lower Pleistocene site at Venta Micena (Orce, Guadix-Baza Basin, Granada, Spain). Geobios.

[72] Campaña I (2016). New interpretation of the Gran Dolina-TD6 bearing *Homo**antecessor* deposits through sedimentological analysis. Sci. Rep..

[73] Pokines JT, Kerbis-Peterhans JC (2007). Spotted hyena (*Crocuta**crocuta*) den use and taphonomy in the Masai Mara National Reserve, Kenya. J. Archaeol. Sci..

[74] Huguet R (2013). Successful subsistence strategies of the first humans in southwestern Europe. Quatern. Int..

[75] Barsky D (2015). Limestone percussion tools from the late Early Pleistocene sites of Barranco León and Fuente Nueva 3 (Orce, Spain). Philos. Trans. R. Soc. B.

[76] Linares-Matás G (2017). Preliminary taphonomical assessment of the macromammalian zooarchaeological assemblage at the late Early Pleistocene site of Cueva Negra del Estrecho del Río Quípar (Murcia, Spain). PESHE.

[77] Rosell R (2017). A resilient landscape at Teixoneres Cave (MIS 3; Moià, Barcelona, Spain): The Neanderthals as disrupting agent. Quatern. Int..

[78] García N, Arsuaga JL (2001). Les carnivores (Mammalia) des sites des Pléistocène ancien et moyen d'Atapuerca (Espagne). Anthropologie.

[79] Linares-Matás GJ, Clark J (2021). Seasonality and Oldowan behavioral variability in East Africa. J. Hum. Evol..

[80] Bunn, H. T. *Meat**eating**and**human**evolution:**Studies**on**the**diet**and**subsistence**patterns**of**Plio-Pleistocene**hominids**in**East**Africa*. Doctoral Thesis. (UC Berkeley, 1982).

[81] Brain CK (1969). The contribution of Namib Desert Hottentots to an understanding of australopithecine bone accumulations. Sci. Pap. Namib Desert Res. Station.

[82] Lyman R (1994). Vertebrate Taphonomy.

[83] Blumenschine RJ (1995). Percussion marks, tooth marks and the experimental determinations of the timing of hominid and carnivore access to long bones at FLK Zinjanthropus, Olduvai Gorge, Tanzania. J. Hum. Evol..

[84] Potts R, Shipman P (1981). Cutmarks made by stone tools from Olduvai Gorge, Tanzania. Nature.

[85] Domínguez-Rodrigo M (2009). A new protocol to differentiate trampling marks from butchery cut marks. J. Archaeol. Sci..

[86] Capaldo SD, Blumenschine RJ (1994). A quantitative diagnosis of notches made by hammerstone percussion and carnivore gnawing on bovid long bones. Am. Antiquity.

[87] López-Cisneros P (2019). The exploitation of hunted resources during the Magdalenian in the Cantabrian Region. Systematisation of butchery processes at Coímbre cave (Asturias, Spain). Quatern. Int..

[88] Domínguez-Rodrigo M (2015). A new methodological approach to the taphonomic study of paleontological and archaeological faunal assemblages: A preliminary case study from Olduvai Gorge (Tanzania). J. Arch. Sci..

[89] Yravedra J, Domínguez Rodrigo M (2009). The shaft-based methodological approach to the quantification of long limb bones and its relevance to understanding hominid subsistence in the Pleistocene: Application to four Palaeolithic sites. J. Quat. Sci..

[90] Yravedra J (2021). Use of meat resources in the Early Pleistocene assemblages from Fuente Nueva 3 (Orce, Granada, Spain). Archaeol. Anthropol. Sci..

[91] López-González F (2006). Deciphering bone depositional sequences in caves through the study of manganese coatings. J. Archaeol. Sci..

[92] Behrensmeyer AK (1978). Taphonomic and ecologic information from bone weathering. Paleobiology.

[93] Olsen SL, Shipman P (1988). Surface modification on bone: Trampling versus butchery. J. Archaeol. Sci..

[94] Maximiano A (2012). Geoestadística y arqueología: Una nueva perspectiva analítico-interpretativa en el análisis espacial intra-site. AnalítiKa.

[95] Boëda E (1990). Identification des chaînes opératoires lithiques du paléolithique ancien et moyen. Paleo.

[96] Petraglia MD, Potts R (1994). Water flow and the formation of early Pleistocene artefact sites in Olduvai Gorge, Tanzania. J. Anthropol. Archaeol..

[97] Lenoble A, Bertran P (2004). Fabric of Palaeolithic levels: Methods and implications for site formation processes. J. Archaeol. Sci..

[98] Bertran P (2012). Particle size distribution of lithic assemblages and taphonomy of Palaeolithic sites. J. Archaeol. Sci..

[99] Hofman JL (1986). Vertical movement of artifacts in alluvial and stratified deposits. Curr. Anthropol..

[100] Stockton ED (1973). Shaw’s Creek Shelter: Human displacement of artefacts and its significance. Mankind.

[101] Hodder I, Orton C (1976). Spatial Analysis in Archaeology.

[102] Benito-Calvo A, de la Torre I (2011). Analysis of orientation patterns in Olduvai Bed I assemblages using GIS techniques: Implications for site formation processes. J. Hum. Evol..

[103] Domínguez-Rodrigo M (2012). Autochthony and orientation patterns in Olduvai Bed I: A reexamination of the status of postdepositional biasing of archaeological assemblages from FLK north. J. Archaeol. Sci..

