# Supplementary material for: Hyaenas and early humans in the latest Early Pleistocene of South-Western Europe
Source: Sci Rep. 2021 Dec 15;11:24036. doi: 10.1038/s41598-021-03547-7 (PMC8674336; doi:10.1038/s41598-021-03547-7)
Supplement: Supplementary file 1 — Supplementary Information. [file 41598_2021_3547_MOESM1_ESM.pdf]

SUPPLEMENTARY INFORMATION

Hyaenas and early humans in the latest Early Pleistocene of South-Western Europe

Gonzalo J. Linares-Matás[1,2, *], Norman Fernández Ruiz[2,3], María Haber Uriarte[2, 3], Mariano López Martínez[2], Michael J. Walker[2,4]

1. St. Hugh's College, University of Oxford. Oxford, OX2 6LE, United Kingdom; *gonzalo.linaresmatas@st-hughs.ox.ac.uk
2. Murcian Association for the Study of Palaeoanthropology and the Quaternary (MUPANTQUAT), Murcia, Spain.
3. Department of Prehistory, Archaeology, Ancient History, Mediaeval History and Historiographical Studies, Faculty of Letters, University of Murcia, Murcia, Spain.
4. Department of Zoology and Physical Anthropology, Faculty of Biology, University of Murcia, Murcia, Spain.

Post-depositional discussion of the bone assemblage from the upper layers of CNERQ Complex 2

The assemblage shows a considerable degree of post-depositional fragmentation, with diagnostically dry fractures present in almost 20% of the assemblage, which further contributes to the high degree of fragmentation of the assemblage. This phenomenon artificially drives up the total number of remains and severely underplays the role of biological agents in relation to bone fracturing, thus resulting in a relatively low percentage of 6.5% for diagnostically green fractures in the assemblage; the origin of fractures in fragments <2cm is often hard to determine with accuracy.

Lying closest to the cave wall, metre-square C0e afforded an interesting pattern in terms of secondary taphonomical alterations, particularly in level 2f. 34.3 % of bones from this square show diagnostic dry fractures (n = 115), in contrast with the percentage of 19.6% for the assemblage as a whole. Furthermore, it also provided over 70% of the bones showing rodent-gnawing (n = 25 for the whole assemblage). In this square, there was also a greater incidence of trampling, non-digestion biochemical alterations, and *in situ* authigenic mineral formation. Displacement of bones towards the end of the cave by non-sedimentary processes (e.g. trampling, rodent accumulation), could have favoured not only their accumulation, but also their subsequent exposure to physico-chemical processes, such as interaction of rainwater and the biocalcarenite of the cave wall, thereby leading to diagenetic alterations and post-depositional fragmentation of bone elements, and secondary mineral precipitation on some bone surfaces. Presence of gnawing marks on generally dry and weathered bone or antler fragments reflects a typical ethological pattern of several rodent species. It would be relevant to study in depth the morphology of the rodent tooth marks in order to discern the most likely species, given that some (e.g. porcupines) that engage with bones in this way also tend to accumulate them, so they could represent another contributing accumulation agent to the palimpsest under study.

The abundance of dry fractures, particularly in square (C0e), which is near the back of the rock-shelter, together with the presence of weathering and rodent gnawing, alongside low levels of biochemical alterations and waterborne polishing, is noteworthy in the upper levels of Complex

2 of Cueva Negra, in stark contrast to the pattern found in other levels. Unit 6 in Complex 3-2, for example, shows a predominance of green fractures in a context of very low to non-existent weathering and high incidence of biochemical and non-abrasive waterborne alterations, in line with the general interpretation of the sedimentation pattern as the result of recurrent, low-energy events that have yielded such a high-resolution sequence. Therefore, it is plausible that the upper levels of Complex 2 could represent a phase of slightly slower sedimentation rates over a non-geologically significant timescale, given the absence of palaeosol formation[1]. This phenomenon, alongside some degree of bioturbation detected at the micromorphological level[1] compatible with the burrowing activities of rodents and also hyaenas in their dens, likely favoured the generation of an archaeological palimpsest.

References

1. Angelucci, D.E. et al. Rethinking Stratigraphy and Site Formation of the Pleistocene Deposit at Cueva Negra del Estrecho del Quípar (Caravaca de la Cruz, Spain). Quaternary Sci Rev **80**, 196-199 (2013).

Figure S1.

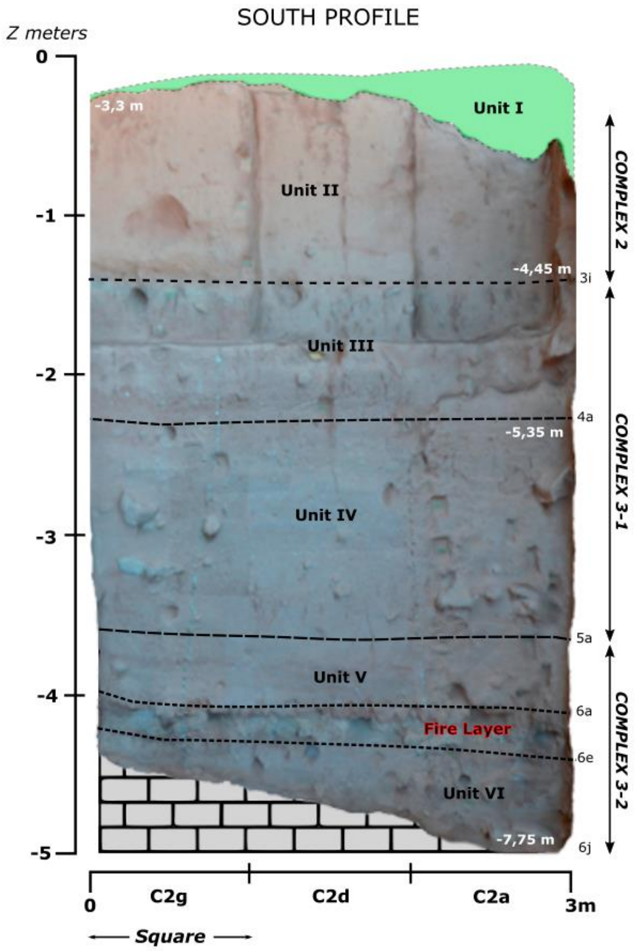

Legend: Stratigraphic profile of Cueva Negra from the South Profile orthophotograph. Note the division and correlation of the sequence into both excavation units and geoarchaeological complexes.

Figure S2.

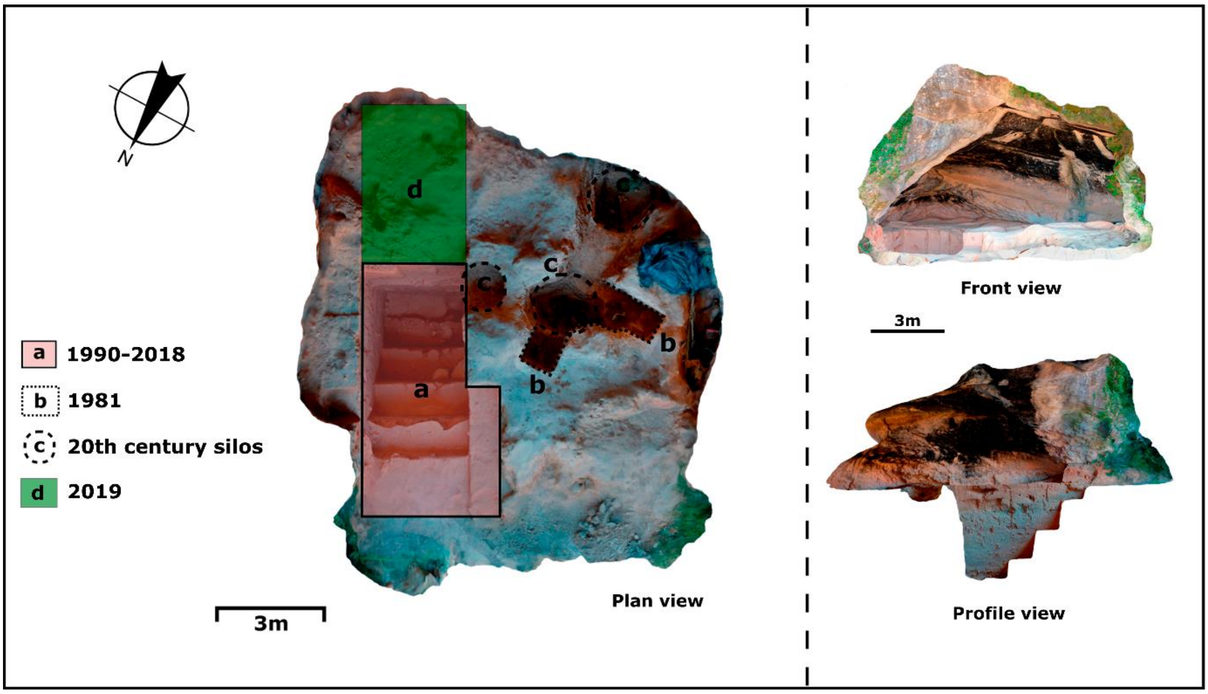

Legend: 3D Cave topography illustrating the different excavated sectors. The assemblage that comprises the bulk of this research comes from the 2019 excavation area (in green). Photogrammetry carried out by Pedro Lucas Salcedo, Inkscape composition by N. Fernández.

Figure S3.

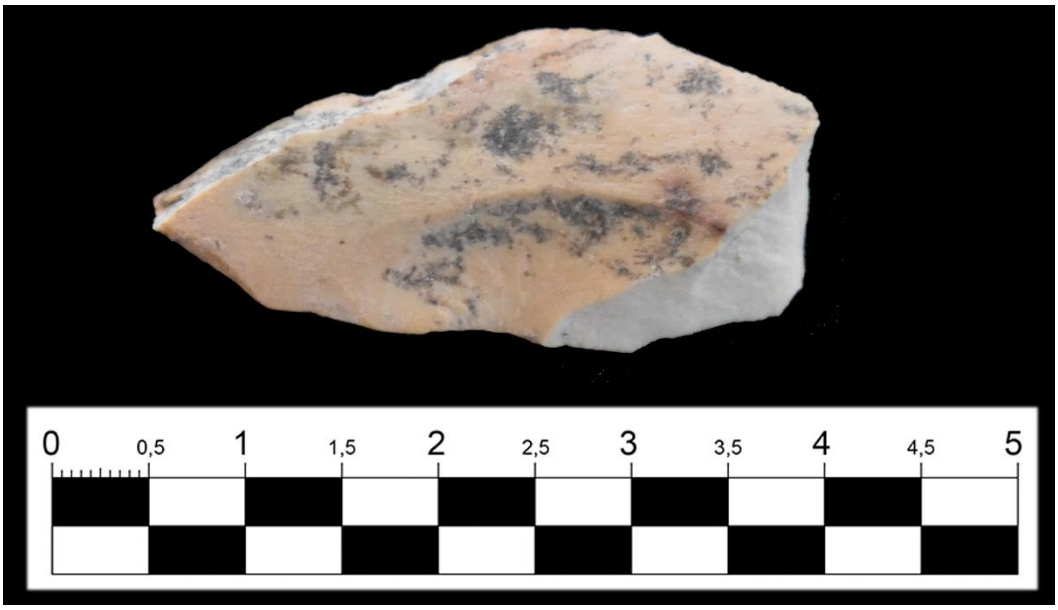

Legend: Fragment of long bone diaphysis from a medium-sized herbivore with an impact negative from the dynamic loading of a percussive action. Photographic composition by Pedro Lucas Salcedo, and reproduced with permission.

Figure S4.

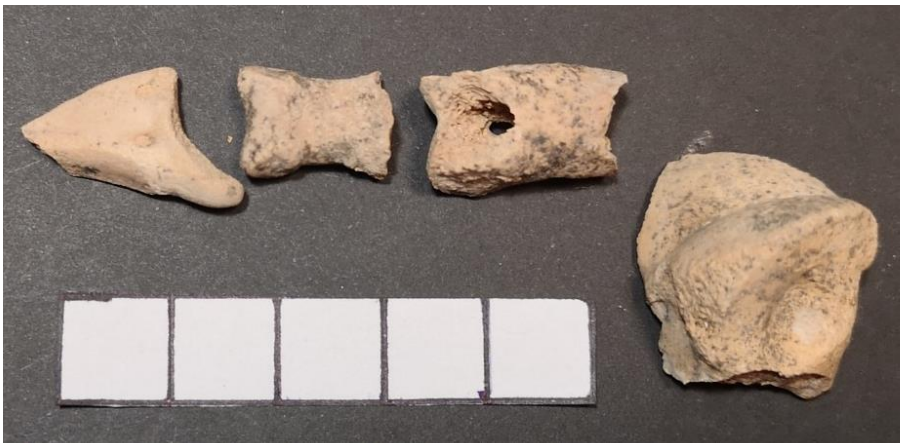

Legend: Phalanges displaying evidence of carnivore damage. Note the unfused proximal epiphysis of the second phalanx effects of digestion on the three associated phalanges of a juvenile cervid (unfused second phalanx, gastric acid pit on first phalanx). The first, second and third phalanges on top were found in spatial association and may well belong to the same individual. Picture taken by G.L.M. using Celestron Photoscan.

Figure S5.

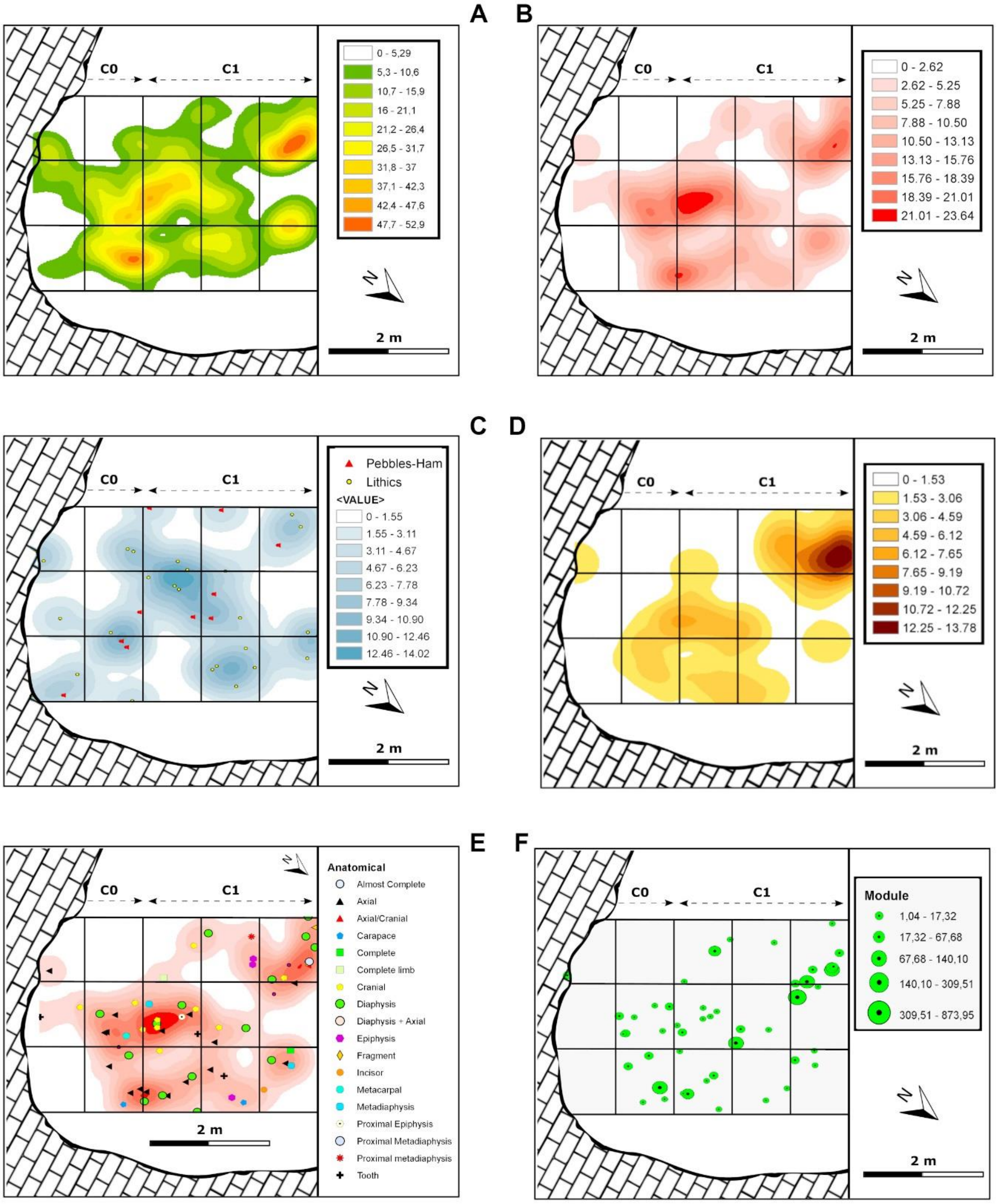

Legend: Spatial distribution analysis of levels 2d, 2e, 2f in sectors C1 and C0. A— PCA Kernel density for the totality of coordinated finds. B— PCA Kernel density for bone elements. C— PCA Kernel density for the totality of lithics and flakes and manuports-hammerstones distribution. D— PCA Kernel density for bones with carnivore marks. E— PCA Kernel density for the totality of bone pieces taking into account anatomical features. F— Distribution of point by modulus (Spatial analysis performed with Arcmap "Spatial Analyst Tools", with visualisation using Inkscape.)

Figure S6.

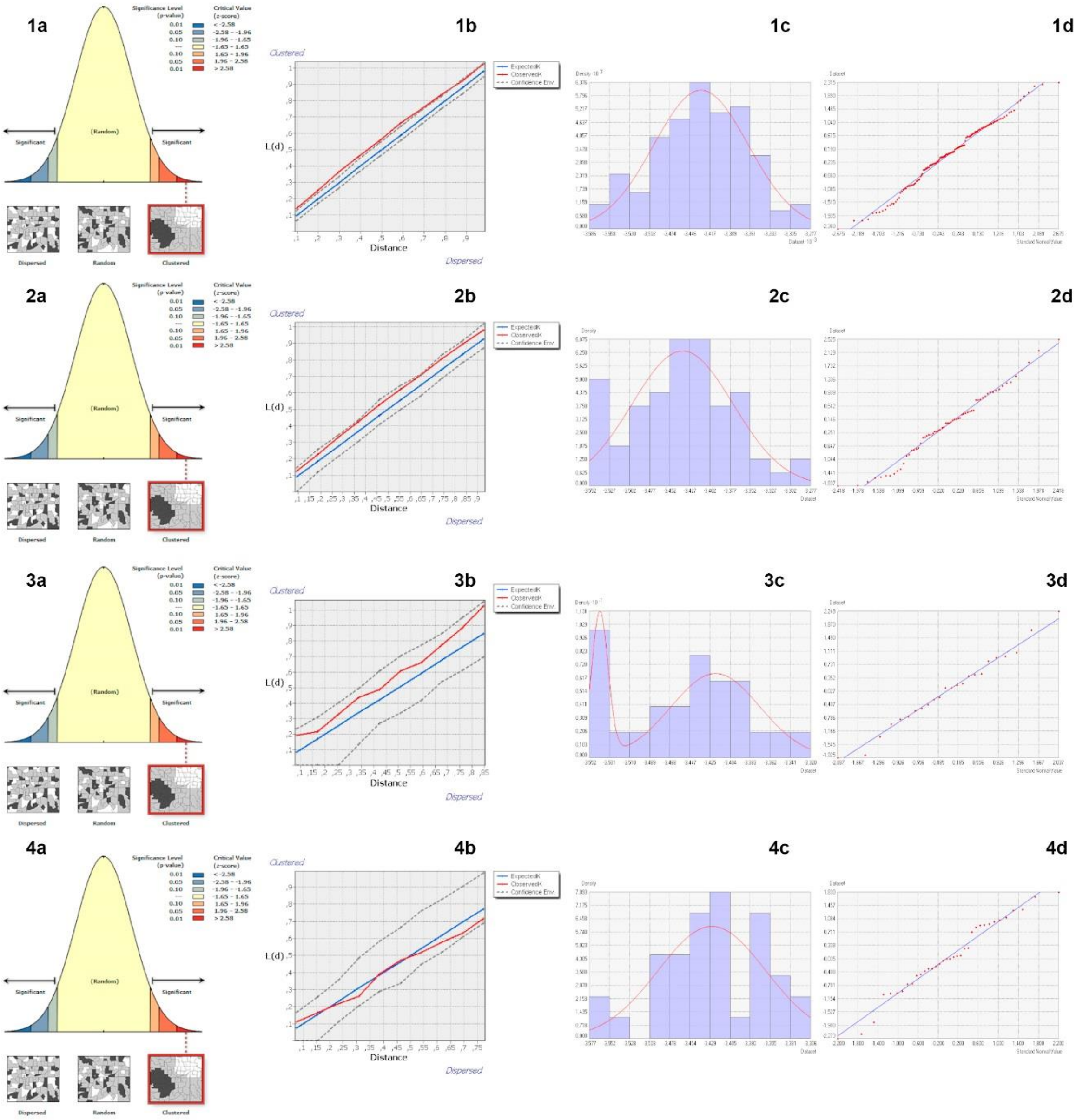

Legend: Spatial statistics (Performed with Arcmap "Spatial Analyst Tools, "Spatial Statistics Tools" and "Geostatistical Analyst Tools").

1a – 4a: Spatial autocorrelation analysis using Moran's I from layers 2d, 2e, and 2f, in sectors C1 and C0 (see table S5). 1a— totality of remains recorded, given the z-score = 21.9525967293, the probability is <1% that the pattern of grouping owes to random assortment. 2a— totality of bone remains, given the z-score = 13.778122, the probability is <1% that the pattern of grouping owes to random assortment. 3a— totality of bone remains showing carnivore marks, given the z-score = 10.2879, the probability is <1% that the pattern of grouping owes to random assortment. 4a— totality of lithic remains recorded from layers 2d, 2e, and 2f, given the z-score = 6.7318, the probability is <1% that the pattern of grouping owes to random assortment.

1b – 4b: Multi-distance spatial cluster analysis using Ripley´s K Function (see table S7). The computed confidence envelope = 999 permutations. Boundary correction method = Ripley

edge correction formula. Number of distance bands = 10. 1b─ totality of remains from layers 2d, 2e, 2f. K-value > HiConfEnv, from which it follows that the spatial clustering is statistically significant. 2b─ totality of bone remains. The K-value exceeds the expected K-value, from which it is inferred that the pattern of spatial clustering is not necessarily of statistical significance. 3b─ totality of remains from layers 2d, 2e, and 2f that present carnivore marks. The K-value exceeds the expected K-value, from which it is inferred that the pattern of spatial clustering is not necessarily of statistical significance. 4b─ totality of lithic remains from layers 2d, 2e, and 2f. The K-value is below the expected K-value, reflecting a more dispersed distribution than expected for a random distribution over medium and long distances.

1c – 4c and 1d – 4d: Frequency graphs of probability density (vertical distribution), histogram and QQplot of standard normality. Statistical analysis was performed with Arcmap "Geostatistical Analyst Tools" and application of Gaussian approximation (see table S6). 1c-1d─ totality of georeferenced finds. The sample shows a normal distribution with a symmetrical unimodal Gaussian curve. 2c-2d─ totality of georeferenced bones. The sample shows a normal distribution with a symmetrical unimodal Gaussian curve, indicating presence of a single population in the sample. 3c-3d─ totality of bones showing carnivore marks. The sample shows a normal distribution for the sample with a bimodal curve indicating that one of the two populations present in the sample has a spread of variation different from the other one; in other words, the two peaks can be interpreted as reflecting both the noteworthy impingement of carnivores and the particularly low variation represented in the left-hand peak. 4c-4d─ totality of georeferenced lithic finds. The sample shows a normal distribution with a unimodal Gaussian curve, indicating presence of a single population in the sample.

Table S1

TAXA	MNI	Juvenile	Subadults	Prime Adults	Old Adults
Bison sp.	1	0	0	1?	0
Caprini (?Capra alba/ ?Hemitragus bonali)	2	1	0	1	0
Crocuta sp.	2	1	1	0	0
Equus cf. *altidens*	1	1	0	0	0
Megaloceros novocartaghiniensis	4	1	2	1	0
Sus scrofa	1	0	1	0	0
Eurotestudo (Testudo) hermanni	4	2	1	1	0
Ursus cf. *deningeri*	1	0	0	0	1
Stephanorhinus cf. *etruscus*	2	1	1	0	0
Total	**18**	**7**	**6**	**4**	**1**

Legend: Taxonomical profile of the faunal assemblage from the upper levels of Complex 2 at Cueva Negra on the basis of MNI. The very high degree of assemblage fragmentation makes this metric the most reliable to assess overall taxonomic species composition in the assemblage, as opposed to MNE/NISP. NISP distribution per size class data is included in Tables 1 and S2.

Table S2

Taphonomical marks	**Very Large Size**	**Large Size**	**Medium Size**	**Small size**	**Indet Size**	**Total**
Pits	20 (47.62%)	48 (26.8%)	57 (30.32%)	23 (30%)	33 (2.98%)	181
Scores	11 (26.19%)	29 (16.2%)	34 (18.08%)	8 (11.25%)	11 (0.99%)	93
Puncture	0	0	1 (0.53%)	0	1 (0.09%)	2
Crenulated edge	0	1 (0.56%)	4 (2.12%)	0	5 (0.45%)	10
Diaphysial cylinder	0	0	0	0	0	0
Furrowing	2 (4.76%)	7 (3.9%)	5 (26.59%)	3 (3.75%)	4 (0.36%)	23
Crushing	1 (2.38%)	0	0	0	2 (0.18%)	3
Pitting	0	1 (0.56%)	1 (0.53%)	1 (1.25%)	0	3
Digestion	1 (2.38%)	28 (15.64%)	40 (21.28%)	13 (16.25%)	66 (7.32%)	163
Licking	0	3 (1.67%)	3 (1.59%)	0	4 (0.36%)	10
Cut-marks	0	22 (12.3%)	7 (3.72%)	2 (2.5%)	9 (0.81%)	40
Percussion marks	0	1 (0.56%)	3 (1.59%)	0	0	4
Burning	0	0	0	0	1 (0.09%)	1
Notches A-B	3 (7.14%)	1 (0.56%)	1 (0.53%)	0	1 (0.09%)	6
Notches C-D	0	6 (3.35%)	7 (3.72%)	1 (1.25%)	0	14
**NR**	42	179	188	80	1107	1596

Legend: Distribution of taphonomic alterations with regards to carcass size in the faunal assemblage from the upper levels of Complex 2 at CNERQ.

Table S3

Taphonomic marks	**Cranial**	**Axial**	**Epiphysis**	**Diaphysis**	**Carpals/ Phalanges**	**Indet**	**Total**
Pits	9 (7.4%)	91 (29.16%)	13 (9.1%)	60 (15.1%)	3 (30%)	5 (0.81%)	181
Scores	2 (1.65%)	44 (14.1%)	5 (3.5%)	39 (9.84)	0	3 (0.49%)	93
Puncture	0	1 (0.32%)	0	0	1 (10%)	0	2
Crenulated edge	0	5 (1.6%)	0	3 (0.75%)	0	2 (0.32%)	10
Diaphysial cylinder	0	0	0	0	0	0	0
Furrowing	4 (3.3%)	10 (3.2%)	4 (2.79%)	2 (0.5%)	1 (10%)	0	21
Crushing	0	3 (0.96%)	0	0	0	0	3
Pitting	0	3 (0.96%)	0	0	0	0	3
Digestion	7 (5.8%)	45 (14.4%)	6 (4.2%)	68 (17.17%)	10 (100%)	12 (1.95%)	148
Licking	1 (0.82%)	3 (0.96%)	2	2 (0.5%)	0	2 (0.32%)	10
Cut-marks	2 (1.6%)	21 (6.7%)	0	11 (2.7%)	0	6 (0.98%)	40
Percussion marks	0	1 (0.32%)	0	3 (0.75%)	0	0	4
Burning	0	0	0	0	0	1 (0.16%)	1
Notches A-B	0	2 (0.64%)	0	4 (1.01%)	0	0	6
Notches C-D	0	7 (2.24%)	0	7 (1.76%)	0	0	14
**NR**	121	312	143	396	10	614	1596

Legend: Distribution of taphonomic alterations with regards to skeletal part in the faunal assemblage from the upper levels of Complex 2 (2c-2f) at CNERQ. Percentages in relation to NR. The limited incidence of biostratinomic alterations on small indeterminate remains reinforces the notion that post-depositional processes played a notable role in the fragmentation of this assemblage.

Table S4

	2c	2d	2e	2f	Total
Pits	17 (11.7%)	54 (9.6%)	67 (11.27%)	43 (14.7%)	181
Scores	9 (5.66%)	23 (3.75%)	48 (7.43%)	13 (4.1%)	93
Puncture	0	0	2	0	2
Crenulated edge	1	3	4	2	10
Diaphysial cylinder	0	0	0	0	0
Furrowing	2	1	7	11	21
Crushing	0	1	2	0	3
Pitting	0	1	2	0	3
Digestion	15 (10.34%)	50 (8.86%)	61 (10.27%)	22 (7.5%)	148
Licking	0	1	8	1	10
Cut-marks	5 (3.1%)	21 (3.43%)	8 (1.24%)	6 (1.89%)	40
Percussion marks	0	3	0	1	4
Burning	0	0	1	0	1
Notches A-B	1	1	4	0	6
Notches C-D	1	10	3	0	14
**NR**	145 (9.1%)	564 (35.3%)	594 (37.2%)	292 (18.27%)	1596

Legend: Distribution of taphonomic alterations with regards to artificial stratigraphic layer in the faunal assemblage retrieved during the 2019 season from the upper levels of Complex 2 at Cueva Negra.

Table S5

Analized Element	Nº Samples	Moran's Index	Expected Index	Variance	z-score	p-value	Dist. Method	Dist. Thresh.	Pattern
Total ID	134	0,8426	-0,0075	0,0015	21,9526	0,0000	Euclidean	0,4110 m	Clustered
Total Bones	64	0,6922	-0,0158	0,0026	13,7781	0,0000	Euclidean	0,6463 m	Clustered
Carnivore Marks	24	0,6352	-0,0434	0,0043	10,2879	0,0000	Euclidean	1,3479 m	Clustered
Lithics	36	0,538	-0,0285	0,0071	6,7318	0,0000	Euclidean	0,7755 m	Clustered

Legend: Statistical data from Moran's spatial autocorrelation index, for the remains from layers 2d, 2e, 2f from sectors C0 and C1. 2019 field season.

Table S6

**Lithics + Bones n=134 Layers: 2d,2e,2f**										**Total Bones n=64 Layers: 2d,2e,2f**									
Average ExpectedK	0,538	*Average ObservedK*	0,596	*Average DiffK*	0,057	*Average LwConfEnv*	0,507	*Average HiConfEnv*	0,585	*Average ExpectedK*	0,509	*Average ObservedK*	0,565	*Average DiffK*	0,055	*Average LwConfEnv*	0,447	*Average HiConfEnv*	0,586
Count ExpectedK	10	*Count ObservedK*	10	*Count DiffK*	10	*Count LwConfEnv*	10	*Count HiConfEnv*	10	*Count ExpectedK*	10	*Count ObservedK*	10	*Count DiffK*	10	*Count LwConfEnv*	10	*Count HiConfEnv*	10
Max ExpectedK	0,979	*Max ObservedK*	1,024	*Max DiffkK*	0,073	*Max LwConfEnv*	0,945	*Max HiConfEnv*	1,028	*Max ExpectedK*	0,926	*Max ObservedK*	0,980	*Max DiffkK*	0,066	*Max LwConfEnv*	0,869	*Max HiConfEnv*	1,017
Min ExpectedK	0,097	*Min ObservedK*	0,140	*Min DiffK*	0,041	*Min LwConfEnv*	0,065	*Min HiConfEnv*	0,129	*Min ExpectedK*	0,092	*Min ObservedK*	0,125	*Min DiffK*	0,032	*Min LwConfEnv*	0	*Min HiConfEnv*	0,146
σ ExpectedK	0,296	*σ ObservedK*	0,294	*σ DiffK*	0,012	*σ LwConfEnv*	0,294	*σ HiConfEnv*	0,302	*σ ExpectedK*	0,280	*σ ObservedK*	0,287	*σ DiffK*	0,011	*σ LwConfEnv*	0,289	*σ HiConfEnv*	0,290
Sum ExpectedK	5,388	*Sum ObservedK*	5,963	*Sum DiffK*	0,575	*Sum LwConfEnv*	5,074	*Sum HiConfEnv*	5,853	*Sum ExpectedK*	5,093	*Sum ObservedK*	5,652	*Sum DiffK*	0,559	*Sum LwConfEnv*	4,476	*Sum HiConfEnv*	5,864
**Carnivore Marks n=24 Layers: 2d,2e,2f**										**Lithics n=36 Layers: 2d,2e,2f**									
Average ExpectedK	0,465	*Average ObservedK*	0,561	*Average DiffK*	0,096	*Average LwConfEnv*	0,301	*Average HiConfEnv*	0,637	*Average ExpectedK*	0,424	*Average ObservedK*	0,405	*Average DiffK*	-0,018	*Average LwConfEnv*	0,320	*Average HiConfEnv*	0,598
Count ExpectedK	10	*Count ObservedK*	10	*Count DiffK*	10	*Count LwConfEnv*	10	*Count HiConfEnv*	10	*Count ExpectedK*	10	*Count ObservedK*	10	*Count DiffK*	10	*Count LwConfEnv*	10	*Count HiConfEnv*	10
Max ExpectedK	0,846	*Max ObservedK*	1,025	*Max DiffkK*	0,179	*Max LwConfEnv*	0,694	*Max HiConfEnv*	1,052	*Max ExpectedK*	0,771	*Max ObservedK*	0,715	*Max DiffkK*	0,033	*Max LwConfEnv*	0,688	*Max HiConfEnv*	0,982
Min ExpectedK	0,084	*Min ObservedK*	0,192	*Min DiffK*	0,046	*Min LwConfEnv*	0	*Min HiConfEnv*	0,236	*Min ExpectedK*	0,077	*Min ObservedK*	0,110	*Min DiffK*	-0,065	*Min LwConfEnv*	0	*Min HiConfEnv*	0,169
σ ExpectedK	0,256	*σ ObservedK*	0,280	*σ DiffK*	0,037	*σ LwConfEnv*	0,263	*σ HiConfEnv*	0,275	*σ ExpectedK*	0,233	*σ ObservedK*	0,209	*σ DiffK*	0,032	*σ LwConfEnv*	0,244	*σ HiConfEnv*	0,277
Sum ExpectedK	4,655	*Sum ObservedK*	5,618	*Sum DiffK*	0,963	*Sum LwConfEnv*	3,011	*Sum HiConfEnv*	6,376	*Sum ExpectedK*	4,242	*Sum ObservedK*	4,054	*Sum DiffK*	-0,187	*Sum LwConfEnv*	3,204	*Sum HiConfEnv*	5,989

Legend: Statistical data of the Multi-distance spatial cluster analysis Ripley´s K Function as applied to all coordinated remains (total), bone elements, bones with carnivore damage, and lithics. Layers 2d, 2e, 2f of sectors C1 and C0; 2019 field season.

Table S7

Type	Samples	Min	Max	Mean	Std. Dev.	Skewness	Kurtosis	1st Quartile	Median	3rd Quartile
Total	134	-3,586	-3,277	-3,430	66,338	-0,12474	2,6523	-3,475	-3,428	-3,381
Total Bone	64	-3,552	-3,277	-3,436	0,063	0,102	2,657	-3,481	-3,434	-3,390
Carnivore Marks	24	-3,552	-3,320	-3,449	0,067	-0,069	2,028	-3,512	-3,434	-3,400
Lithics	36	-3,557	-3,306	-3,426	0,066	-0,282	2,653	-3,468	-3,429	-3,367

Legend: Statistical data from the Gaussian distribution and probabilistic frequency analysis for all coordinated remains (total), bone elements, bones with carnivore damage, and lithics. Layers 2d, 2e, 2f; 2019 field season.

Table S8

Square	Mn 0	Mn 1	Mn 2	Mn 3	Mn Coefficient
C1h	28	55	31	26	1.393
C1g	7	29	17	9	1.452
C1e	20	70	62	67	1.804
C1d	11	65	54	19	1.544
C1b	3	27	27	33	2.000
C1a	4	24	32	32	2.000
C0i	7	15	11	15	1.708
C0f	37	79	19	13	1.054
C0e	45	132	103	42	1.441
C0c	7	30	44	21	1.775
**Total**	**169**	**526**	**400**	**277**	**1.572**

Legend: Spatial distribution of Mn alterations in the CNERQ upper Complex 2 bone assemblage (only available for layers 2d-2f, n = 1372). Mn 0 represents no Manganese staining, whereas Mn 3 represents dark and almost complete Manganese staining of the bone. The “Mn coefficient represents the ponderated average of Mn staining to facilitate inter-square comparisons, as calculated by the formula (Mn0*0+Mn1*1+Mn2*2+Mn3*3)/(Mn0+Mn1+ Mn2+ Mn3).

Table S9

Square	Weath 0	Weath 1	Weath 2+	Biochem 0	Biochem 1	Biochem 2	Trampling 0	Trampling 1+
C1h	111	25	1	101	32	4	135	2
C1g	57	5	0	57	5	0	57	5
C1e	166	54	2	201	18	3	224	3
C1d	125	23	1	123	22	4	147	2
C1b	73	17	0	79	11	0	89	1
C1a	70	21	1	73	19	0	91	1
C0i	46	2	0	38	10	0	48	0
C0f	135	11	2	124	24	0	141	7
C0e	289	31	2	204	112	6	311	11
C0c	94	8	0	86	15	1	101	1
**Total**	**1166**	**197**	**9**	**1138**	**216**	**18**	**1339**	**33**

Legend: Spatial distribution and intensity of weathering, biochemical alterations, and trampling in the CNERQ upper Complex 2 bone assemblage (only available for layers 2d-2f, n = 1372).
